# Genetic targeting of adult Renshaw cells using a *Calbindin 1* destabilized Cre allele for intersection with *Parvalbumin* or *Engrailed1*

**DOI:** 10.1038/s41598-021-99333-6

**Published:** 2021-10-06

**Authors:** Alicia R. Lane, Indeara C. Cogdell, Thomas M. Jessell, Jay B. Bikoff, Francisco J. Alvarez

**Affiliations:** 1 Department of Physiology, Emory University, Atlanta, GA 30322 USA; 2 Department of Developmental Neurobiology, St. Jude Children’s Research Hospital, Memphis, TN 38105 USA; 3 Department of Biochemistry and Molecular Biophysics, Columbia University, New York, NY 10032 USA

**Keywords:** Neural circuits, Neuroscience, Motor control, Spinal cord

## Abstract

Renshaw cells (RCs) are one of the most studied spinal interneurons; however, their roles in motor control remain enigmatic in part due to the lack of experimental models to interfere with RC function, specifically in adults. To overcome this limitation, we leveraged the distinct temporal regulation of *Calbindin* (*Calb1*) expression in RCs to create genetic models for timed RC manipulation. We used a *Calb1* allele expressing a destabilized Cre (dgCre) theoretically active only upon trimethoprim (TMP) administration. TMP timing and dose influenced RC targeting efficiency, which was highest within the first three postnatal weeks, but specificity was low with many other spinal neurons also targeted. In addition, dgCre showed TMP-independent activity resulting in spontaneous recombination events that accumulated with age. Combining *Calb1*-dgCre with *Parvalbumin* (*Pvalb*) or *Engrailed1* (*En1*) Flpo alleles in dual conditional systems increased cellular and timing specificity. Under optimal conditions, *Calb1-dgCre/Pvalb-Flpo* mice targeted 90% of RCs and few dorsal horn neurons; *Calb1-dgCre/En1-Flpo* mice showed higher specificity, but only a maximum of 70% of RCs targeted. Both models targeted neurons throughout the brain. Restricted spinal expression was obtained by injecting intraspinally AAVs carrying dual conditional genes. These results describe the first models to genetically target RCs bypassing development.

## Introduction

Renshaw cells (RCs) are inhibitory spinal interneurons that synapse onto motoneurons (MNs) and receive direct input from recurrent collaterals of motor axons as they exit the spinal cord^1–4^. This MN-RC recurrent circuit, discovered in 1946^4^, is the oldest known inhibitory circuit in the mammalian CNS and is presumed to exert rapid feedback control of MN activity. Despite its simplicity and longstanding literature presence, its exact function is still debated. Detailed analyses of RC connections, input/output properties and the dynamic behavior of RC synapses on MNs and Ia inhibitory interneurons led to several hypotheses about possible RC functions that were critically reviewed in 1996^5^. To this day, these hypotheses remain unchanged and largely untested^6,7^, in part due to the inability to specifically manipulate RCs and isolate their activity from other network elements during motor actions. Because of these limitations, several studies have instead opted to interrogate RCs through computational models^8–12^. However, these approaches necessarily rely on assumptions about unknown parameters, including the exact synaptic connectivity between RCs and motor pools and the full complement of RC inputs and outputs^6^. To better understand the function of this circuit and validate computational models, it is necessary to perform experiments that selectively target RCs to precisely reveal their full connectome and alter their activity specifically during motor actions in vivo.

Recent advances in molecular characterization of RCs suggest possible genetic approaches to specifically label and manipulate RCs. Renshaw cells uniquely receive strong cholinergic inputs and display high postsynaptic nicotinic sensitivity^6^, mediated in part by expression of the α2 nicotinic receptor subunit (*Chrna2*)^13^. This property was used to develop a *Chrna2-Cre* bacterial artificial chromosome (BAC) transgenic line—the only mouse model for RC targeting published to date^14,15^. These mice were used to delete the vesicular inhibitory amino acid transporter (VIAAT), thereby preventing throughout development inhibitory neurotransmission from RCs and other *Chrna2* expressing cells in brain and spinal cord^14^. No significant effects were detected in motor function during in vitro fictive locomotion or whole-animal locomotion. The authors concluded that compensatory changes during development possibly obscured any functional deficits due to RC silencing. This highlights the need for novel strategies to target RCs after the maturation of spinal networks is completed. However, it should be also noted that analyses to confirm synaptic silencing of RCs or the recurrent inhibitory circuit were not performed in this study^14^ and therefore alternative explanations are also possible.

Molecular and developmental studies of RCs suggest several alternative genetic strategies for manipulating RCs with greater temporal control. RCs are a type of V1 interneuron that express the transcription factors engrailed-1 (*En1,* defining all V1 interneurons), musculoaponeurotic fibrosarcoma oncogene homolog A and B (*Mafa*, *Mafb*), as well as *Oc1*, *Oc2*, and *Foxd3*^16–19^. In addition, all RCs express calbindin (*Calb1*) and many express parvalbumin (*Pvalb*) and/or calretinin (*Calb2*)^16^. Each of these genes is developmentally regulated, offering temporal windows for RC targeting. For example, *En1* and *Mafb* are expressed in many embryonic V1 interneurons, and while *En1* is downregulated in postnatal RCs, these same RCs maintain *Mafb* expression^17–19^. Similarly, early widespread *Calb1* expression is downregulated postnatally, but specifically maintained by mature/adult RCs within the ventral horn and the V1 group^20^. In contrast, *Pvalb* expression in spinal interneurons, including RCs, only begins after P10 and becomes widespread later^20^. None of these genes are unique to RCs, necessitating combinatorial approaches to increase targeting specificity. We thus explored the temporal dynamics and combinatorial expression of these genes to target RCs in their mature state.

To bypass development, we took advantage of *Calb1-2A-dgCre-D* mice (JAX#023531, referred to as *Calb1*-dgCre) that express a destabilized version of Cre (dgCre) dependent on *Calb1* regulatory sequences^21^. dgCre consists of an N-terminal fusion protein of Cre with the first 159 amino acids of the *Escherichia coli* dihydrofolate reductase (ecDHFR), directing Cre to proteosomal degradation and preventing nuclear translocation and recombination unless degradation is blocked with trimethoprim (TMP)^22^. We analyzed optimal TMP dosage and timing to target RCs using the *Calb1*-dgCre allele, validating labeling specificity using a combination of localization criteria and *Mafb* gene expression using a *Mafb-GFP* animal^23^. To increase specificity, we vetted calretinin (CR, *Calb2* gene) and parvalbumin (PV, *Pvalb* gene) against *Calb1-dgCre* mice. Intersectional designs combining *Calb1-dgCre* mice with a *Pvalb-2A-FlpO-D* animal^24^ (JAX#022730, referred to as *Pvalb*-Flpo) resulted in over 90% targeting of RCs and restricted targeting to after P10. However, a few dorsal horn cells, as well as significant groups of brain neurons were also targeted. To more specifically target the lumbar spinal cord, we show that dual conditional transgenes can be effectively introduced into RCs by local intraspinal delivery of AAV9 vectors. To restrict targeting to ventral inhibitory interneurons, we characterized a new *En1*-Flpo knock-in animal to focus *Calb1*-dgCre targeting to V1 interneurons. We found 60–70% RC targeting with this combination. These results describe the first models for in vivo adult RC targeting. We discuss the advantages and disadvantages of each model with the objective that these models, or variations thereof, can be adopted to accelerate discovery of RC functions.

## Results

### Identification criteria for Renshaw cells and the Renshaw cell area

**Figure 1:**
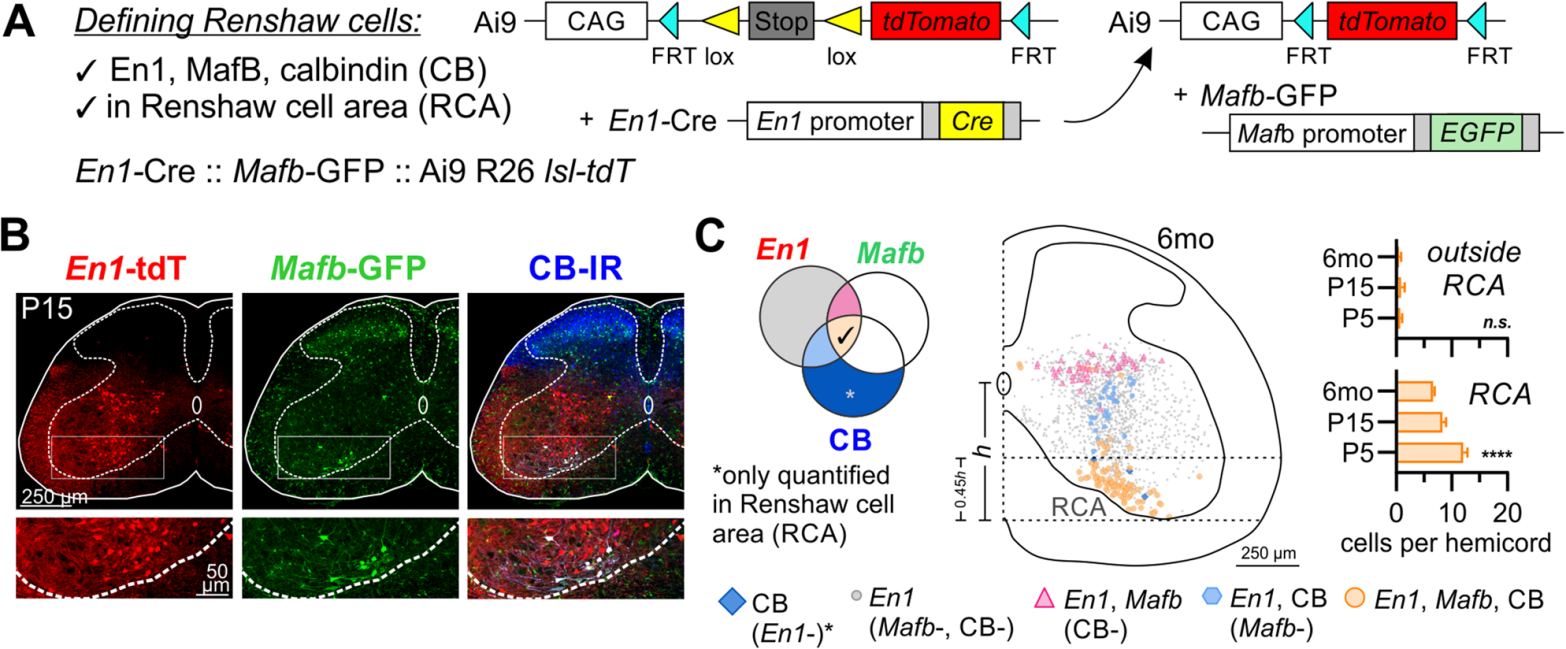
Genetic strategy to identify and define Renshaw cells. **(A)** Triple transgenic *En1-*Cre :: *Mafb-*GFP :: Ai9 R26 *lsl-tdT* animals label *Mafb-*expressing cells with GFP and the V1 lineage with tdTomato (tdT). (**B)** 2D projection of a triple fluorescence confocal image stack (50 µm thick) showing *En1*-tdT (V1 interneurons, red), *Mafb-*GFP (green), and calbindin immunoreactivity (CB-IR, blue) at P15. (**C)** Venn diagram illustrates co-localization possibilities between *En1*-tdT, *Mafb-*GFP, and CB-IR; colors match cell plots obtained from a 6-month old animal (12 superimposed 50 µm thick sections). V1-MafB cells appear as distinct dorsal and ventral groups; the ventral group is CB-IR and corresponds to Renshaw cells. The Renshaw cell area (RCA) was anatomically defined as the bottom 45% of the deep ventral horn. This region includes the vast majority of triple-labeled cells (● orange circles); less than one triple-labeled cell per hemicord was observed outside this region across all time points. Graphs show quantification of triple-labeled cells at P5, P15, and 6 months of age inside or outside the RCA (n = 2–4 animals per group, errors bars = S.D.; see Supplemental Table 3). Within the RCA, 98% of CB-IR cells co-express *En1*-tdT and *Mafb-*GFP by P15. Asterisks indicate significance level after One-Way ANOVAs across all conditions. ****p < 0.0001, *n.s.* non-significant. For pair-wise comparisons and details of statistics, refer to Supplemental Table 3.

To determine whether genetically targeted cells across different ages are Renshaw cells (RCs), we validated criteria for RC identification that are independent of age or spinal cord size (Fig. 1). Analyses focused on lumbar 4 and 5 segments (L4, L5). We used *En1-*Cre*/* + :: *Mafb-*GFP*/* + :: Ai9 R26 *lsl-tdT/* + animals (Fig. 1A) to define RCs as triple-labeled calbindin-immunoreactive (CB-IR) V1-MafB cells^25^ (Fig. 1B). At P5, P15, and 6 months of age, 6–7% of V1 interneurons (*En1*-tdT) are positive for *Mafb-*GFP and CB-IR. At all ages, 90–95% of these triple-labeled cells are found in a region we defined as the Renshaw cell area (RCA; Fig. 1B,C, see methods) and that occupies the bottom 45% of the ventral horn in these lumbar segments (Fig. 1C). In the RCA, 98–99% of CB-IR cells are V1-derived and express *Mafb-*GFP at P15 and 6 months of age; however, at P5 this percentage is only 74%. Thus, while there are significant numbers of CB-IR cells in the RCA at P5 that are *not* RCs, CB-IR cells in the RCA are almost exclusively RCs after P15. This is consistent with the progressive downregulation from P5 to P60 of CB-IR in ventral horn cells, other than RCs (Supplemental Fig. S1), which we previously reported^20^.

### Targeting Renshaw cells using *Calb1-*dgCre mice

**Figure 2:**
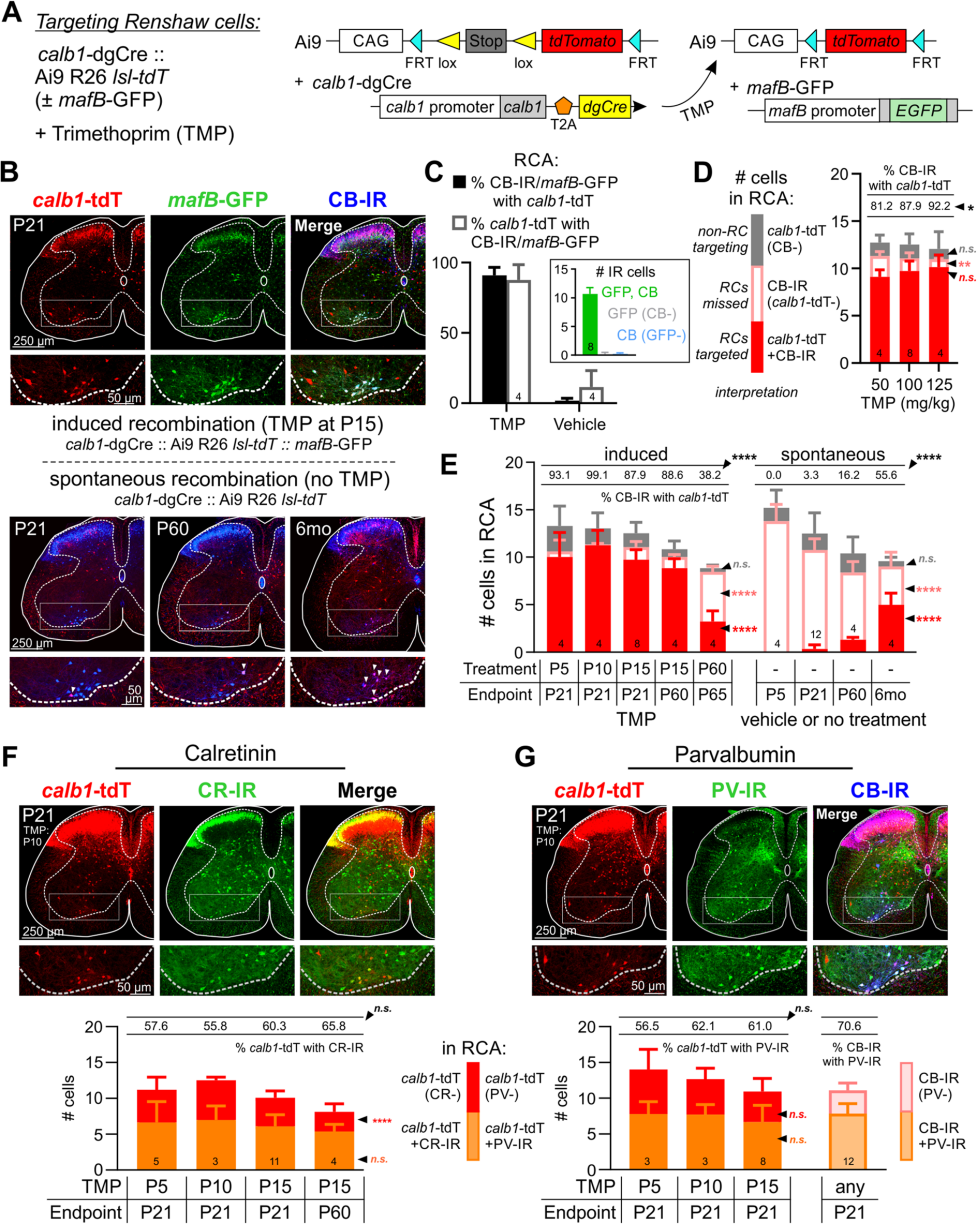
Genetic targeting of Renshaw cells with *Calb1*-dgCre.** (A)** The *Calb1*-dgCre :: Ai9 R26 *lsl-tdT* system labels with tdT interneurons expressing calbindin at the time of TMP administration. The *Mafb-*GFP allele reports cells expressing *MafB*. (**B)** Confocal image (50 µm thick section) showing *Mafb*-GFP and CB-IR in *Calb1*-tdT interneurons. Top: TMP-induced recombination at P15 analyzed at P21 (*Calb1*-tdT, red; *Mafb-*GFP, green; CB-IR, blue). Bottom: Spontaneous recombination at P21, P60, and 6 months in the absence of TMP (bottom panel). (**C)** Colocalization between CB-IR, *Mafb-*GFP, and *Calb1*-tdT within the RCA at P21 following injection of TMP or vehicle at P15. Total cell numbers per RCA are summarized in Supplemental Table 4. (**D,E)** CB-IR colocalization with *Calb1*-tdT in the RCA of *Calb1*-dgCre :: Ai9 R26 *lsl-tdT* mice with different (**D**) TMP doses or (**E**) with or without TMP (indicated by a dash: -) at different injection times (treatment) and time of analysis (endpoint). Color code used in stacked histogram shown in the left. (**D)** There is a small but significant increase in RC targeting with increased TMP dose (see Supplemental Table 5). (**E)** RC targeting (red bars) is significantly reduced when TMP injections are done in the adult (P60), while off-target labeling (grey bars) diminishes with injection age. Spontaneous labeling increases with age (see Supplemental Tables 6–7, Supplemental Fig. S2). (**F,G)** Colocalization of *Calb1*-tdT genetic labeling with calretinin and parvalbumin immunoreactivities in *Calb1*-dgCre :: Ai9 R26 *lsl-tdT* animals at P21 and P60 after TMP administration at P5, P10, P15, or P60. Top: Confocal images (50 μm thick) for (**F**) calretinin (CR-IR) or (**G**) parvalbumin (PV-IR) and calbindin (CB-IR) (*Calb1*-tdT: red; CR-IR or PV-IR: green; CB-IR: blue). Bottom: Quantification in the RCA of *Calb1*-tdT cells and colocalization with (**F**) CR-IR or (**G**) PV-IR and CB-IR (see Supplemental Table 6, Supplemental Fig. S3). In all graphs, error bars = S.D.; and asterisks indicate significance level after One-Way ANOVAs across all conditions. *p < 0.05; **p < 0.01; ***p < 0.001; ****p < 0.0001, *n.s.* non-significant. For pair-wise comparisons and details of statistics refer to the indicated supplemental figures and tables.

Using the above criteria, we analyzed the specificity and efficiency of RCs targeting at different developmental time points using *Calb1-*dgCre mice crossed to Ai9 R26-tdTomato reporter mice (Fig. 2). In these animals, trimethoprim (TMP) administration sets the timing of Cre recombination and tdTomato (tdT) labeling in cells expressing *Calb1* (Fig. 2A). *Calb1*-tdT cells are visible throughout the spinal cord by 24 h after TMP injection with expression plateauing at 36 h (Supplemental Fig. S1). To validate RC targeting efficiency we introduced the *Mafb-*GFP allele in these animals (*Calb1*-dgCre*/* + :: *Mafb-*GFP*/* + :: Ai9 R26 *lsl-tdT/* + ; Fig. 2A), injected 100 mg/kg TMP or vehicle (DMSO/saline solution) at P15, and analyzed the results at P21 (Fig. 2B,C). Within the RCA, 87.8% of genetically labeled *Calb1*-tdT cells expressed both CB-IR and *Mafb-*GFP and 89.1% of these *Calb1*-tdT cells were CB-IR. A high proportion of RCs are labeled in this model; 88.1% of CB-IR cells and 90.7% of CB-IR/*Mafb-*GFP cells in the RCA were *Calb1*-tdT (Fig. 2C). To validate TMP dose, we injected *Calb1*-dgCre*/* + :: Ai9 R26 *lsl-tdT/* + mice with 50, 100, or 125 mg/kg TMP at P15. TMP dose had a modest but significant effect on the percentage of RCs expressing *Calb1*-tdT (p = 0.0132, one-way ANOVA; Fig. 2D, Supplemental Table 3, Supplemental Fig. S2). This percentage differed significantly only between 50 and 125 mg/kg (81.2% vs. 92.2%; p = 0.0126, post-hoc Bonferroni test). We used 100 mg/kg TMP in subsequent experiments as it was not significantly different from 125 mg/kg, which requires higher DMSO concentrations to administer.

Next, we varied the time at which we induced Cre recombination with TMP (Fig. 2E, Supplemental Fig. S2, Supplemental Table 4). Renshaw cell targeting was analyzed at P21 after TMP injections at P5, P10 or P15 or at two months after injections at P15 or P60. Genetic labeling after TMP injections at P5, P10 and P15 was similarly efficient, with 87.9% to 99.1% of RCs expressing *Calb1*-tdT. In contrast, significantly fewer RCs expressed *Calb1*-tdT when recombination was induced at P60 (p < 0.0001, post-hoc Bonferroni compared to all other time points). Comparing RCs at P21 or P60 after P15 TMP injections revealed no significant effect of age of analysis, suggesting stable labeling of RCs after TMP-induced Cre recombination.

Many non-RCs throughout the spinal cord are also targeted in animals treated with TMP (Fig. 2B, top). This was expected given the widespread expression of *Calb1*. Most *Calb1-*tdT cells were in the dorsal horn and many lacked CB-IR at the time of analysis. In the RCA, a small number of *Calb1*-tdT cells without CB-IR were also found. Their number was larger following TMP at P5 and then progressively decreased with TMP injection age, although the trend did not reach significance (Fig. 2E grey bars, Supplemental Fig. S2, Supplemental Table 4). CB-immunonegative *Calb1*-tdT + cells are best explained by calbindin downregulation after TMP administration. In addition, we observed significant numbers of *Calb1*-tdT cells in the absence of TMP, suggesting “leakiness” in the genetic system with some dgCre escaping effective degradation (Fig. 2B, bottom). Untimed TMP-independent labeling, which we refer to as spontaneous recombination, is variable between animals and spinal cord sides. It is also not uniform across cell types and is minimal in RCs until two months of age (Fig. 2C; Supplemental Table 2). *Calb1*-tdT cells in the absence of TMP increased with age, as expected if spontaneous recombination events accumulate with time. Before P21, TMP-independent *Calb1*-tdT cells are mostly in the dorsal horn, but spread to the ventral horn at later ages. By 6 months, TMP-independent *Calb1*-tdT labeling increases dramatically, with *Calb1*-tdT neurons scattered throughout the dorsoventral axis of the spinal cord. Moreover, we observed tdT + sensory axons in dorsal roots and dorsal columns. These axons innervated spinal laminae III and IV, suggestive of cutaneous mechanoreceptors. Notably, spontaneous recombination in RCs is almost absent before P60: 0% of RCs at P5; 3.2% at P21, 16.2% at P60, and 55.6% at 6 months (Fig. 2E, Supplemental Fig. S2; Supplemental Table 5). The percentage of RCs labeled at 6 months and P60 was greater than at prior time points (p ≤ 0.0001, post-hoc Bonferroni tests). In summary, targeting RCs with the *Calb1*-dgCre allele is highly efficient when timed in the first three weeks of life with TMP; however, it lacks specificity, with many other spinal interneurons targeted by either TMP-dependent (timed) or TMP-independent (untimed) activity of *Calb1*-dgCre.

Previous studies indicate that intersection of *Calb1-*dgCre with other calcium buffering proteins expressed by postnatal RCs^16^ might increase specificity while retaining accurate temporal control of RC targeting. Thus, we tested for expression of calretinin (CR) or parvalbumin (PV) in *Calb1*-tdT RCs. Mice injected with TMP at P5, P10, or P15 were analyzed at P21 for CR or PV immunoreactivity. An additional P60 timepoint after TMP injection at P15 was included for CR. Across all conditions, 56–66% of *Calb1*-tdT cells were CR-IR (Fig. 2F, ANOVA, p = 0.5443; Supplemental Fig. S3, Supplemental Table 6) and 57–62% of *Calb1*-tdT cells were PV-IR with no significant differences according to TMP injection age (Fig. 2G, ANOVA, p = 0.8490; Supplemental Fig. S3, Supplemental Table 6). All *Calb1*-tdT RCs with PV-IR were also CB-IR (Fig. 2G). Thus, co-expression of *Calb1* with either *Calb2* (CR gene) or *Pvalb* (PV gene) should target similar numbers of RCs at P21. The possibility of increased PV expression at later time points was not possible to characterize with our antibodies as PV-IR accumulates in the spinal cord postnatally and after P21 immunolabeling is rather diffuse and has limited cell resolution^20^.

### Dual-conditional targeting of Renshaw cells using *Calb1-*dgCre* :: Pvalb-*Flpo animals

**Figure 3:**
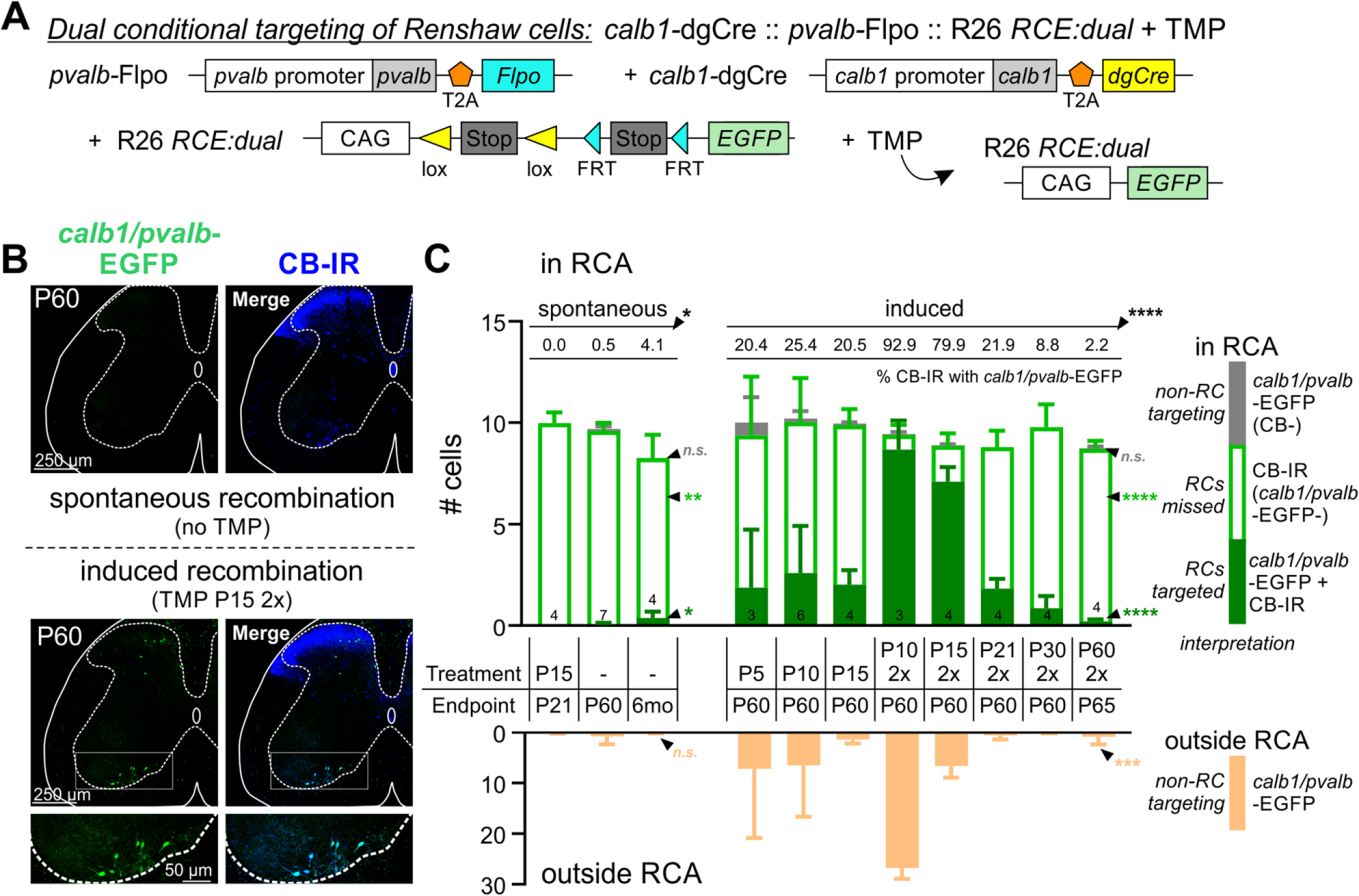
Genetic targeting of Renshaw cells by the intersection of *Calb1*-dgCre and *Pvalb*-Flpo.** (A)** Schematic of the *Calb1*-dgCre :: *Pvalb*-Flpo :: R26-*RCE-dual-EGFP* genetic system. Genetic targeting occurs in cells that excise both stop codons in the R26-*RCE:dual-EGFP* reporter by expressing calbindin at the time of TMP administration and parvalbumin any time prior to age of analysis. (**B)** Confocal image stacks (50 µm thick sections) comparing lack of spontaneous recombination labeling (top) or labeling after two TMP injections at P15 and analysis at P60 (bottom) (*Calb1/Pvalb*-EGFP, green; CB-IR, blue). Most RCs (higher mag panels) and a few dorsal horn cells express EGFP and CB-IR after TMP administration. (**C)** Quantification of *Calb1/Pvalb* -EGFP inside and outside the RCA (top and bottom bars, respectively) after TMP or vehicle injections at different time points from P5 to P60 or no injections. The largest percentage of RC labeling occurs after 2 doses of TMP at P10 or P15. The number of non-RCs targeted diminishes after P15 injections compared to P10. In this intersectional *Calb1/Pvalb* model, there is negligible spontaneous labeling at P60, either in the RCA or outside the RCA. (Error bars = S.D.; asterisks in all graphs indicate significance level after One-Way ANOVAs across all conditions. *p < 0.05; **p < 0.01; ***p < 0.001; ****p < 0.0001, *n.s.* non-significant. For pair-wise comparisons and details of statistics and analyses, see Supplemental Tables 7–9 and Supplemental Fig. S4).

To determine whether an intersectional genetic approach might target Renshaw cells with increased specificity, we took advantage of the availability of animals carrying a *Pvalb1-*Flpo allele to generate *Calb1* dgCre*/* + *:: Pvalb* Flpo*/* + *::* R26 *RCE:dual-EGFP/* + animals, in which EGFP expression requires activity of both recombinases (Fig. 3A)^25^. Compared to *Calb1-*dgCre animals, the number of genetically targeted cells in the absence of TMP is drastically reduced at P21, P60, and 6 months (Fig. 3B top, Fig. 3C, Supplemental Fig. S4, Supplemental Tables 7 and 8). This suggests that spontaneous *Calb1*-dgCre recombination mostly occurs in cells that do not express *Pvalb*-Flpo and genetic intersection with *Pvalb* removes most of these cells.

Next, we analyzed TMP-induced recombination in RCs at 2 months of age following TMP administration at P5, P10, or P15. We expected to target at least 50% of RCs based on PV-IR colocalization at P21 (Fig. 2G); however, only 20%-25% of RCs were *Calb1/Pvalb-*EGFP with no significant differences among injection dates, suggesting TMP stabilization of Cre in this context might be suboptimal (Fig. 3C, Supplemental Fig. S4, Supplemental Table 9). We therefore explored whether two TMP doses separated by 48 h (P10) or 24 h (P15, P21, P30, or P60) could increase RC coverage. Double injections at P10 and P15 labeled 92.9% and 79.9% of RCs, respectively (Fig. 3B, bottom), with no significant difference between the two conditions (p > 0.9999, Bonferroni test; Supplemental Fig. S4, Supplemental Table 9). Consistent with *Calb1*-dgCre mice, the effectiveness of RC targeting decreased when TMP is administered at later ages: 15.8% of RCs were *Calb1*-tdT after double injections at P21, 3.3% at P30, and 0.8% at P60 (p < 0.0001 for all pair-wise Bonferroni comparisons; Supplemental Table 9).

The higher specificity of the *Calb1*/*Pvalb* intersection was best visualized in *Calb1-*dgCre *:: Pvalb-*Flpo animals with two different reporters in the R26 locus: one allele carrying the *RCE:dual-EGFP*, and the other the Ai9 Cre-dependent *lsl-tdT* (Supplemental Fig. S5). In the Ai9 line, the tdT cassette is removed by Flpo recombination; thus, cells expressing both *Calb1* and *Pvalb* are only EGFP-labeled, while cells that express only *Calb1* are tdT-labeled. After a double injection of TMP at P10, tdT labeling at P60 was widespread, while EGFP labeling was restricted to RCs and a few dorsal horn neurons (Supplemental Fig. S5). Surprisingly, some RCs were dual-labeled with tdT and EGFP. This might be explained by late activation of the *Pvalb* promoter and lingering tdT protein, which can be retained by neurons for at least two weeks after switching off expression^43^. Together with our previous estimate that 57–62% of *Calb1-*tdT RCs were PV-IR at P21 (Fig. 2G), this suggests that late upregulation of *Pvalb* in some RCs might explain the larger than expected percentage of RCs targeted at P60 (89% to 97%). Consistent with this, only 72% of RCs were labeled at P21 in animals after similar dual TMP injections at P10 (n = 2 animals). Therefore, RCs continue to upregulate *Pvalb* between P21 and P60, increasing coverage of RCs with age of analysis under the dual condition of *Calb1* and *Pvalb* expression.

In all TMP injection/analysis dates analyzed, there were negligible numbers of *Calb1/Pvalb-*EGFP cells in the RCA that were not RCs (CB-IR negative; Fig. 2C, Supplemental Fig. S4). The number of *Calb1/Pvalb*-EGFP cells outside the RCA was also dramatically reduced, but some dorsal horn cells were still labeled. Remarkably, there was a significant difference between P10 and P15 (26.8 ± 2.1 vs. 6.6 ± 2.3 dorsal horn cells per hemicord ± S.D.; p = 0.0156 Bonferroni multiple comparisons, Supplemental Table 8), despite both targeting similar numbers of RCs. Double TMP injections at P15 therefore increased RC targeting specificity with no significant reduction in efficiency. In conclusion, *Calb1-*dgCre and *Pvalb-*Flpo intersection targets almost all RCs by P60 when two TMP doses are injected between P10 and P15, but the later dual injections result in greater specificity.

### Dual-conditional approach with *Calb1-*dgCre* :: En1-*Flpo animals

**Figure 4:**
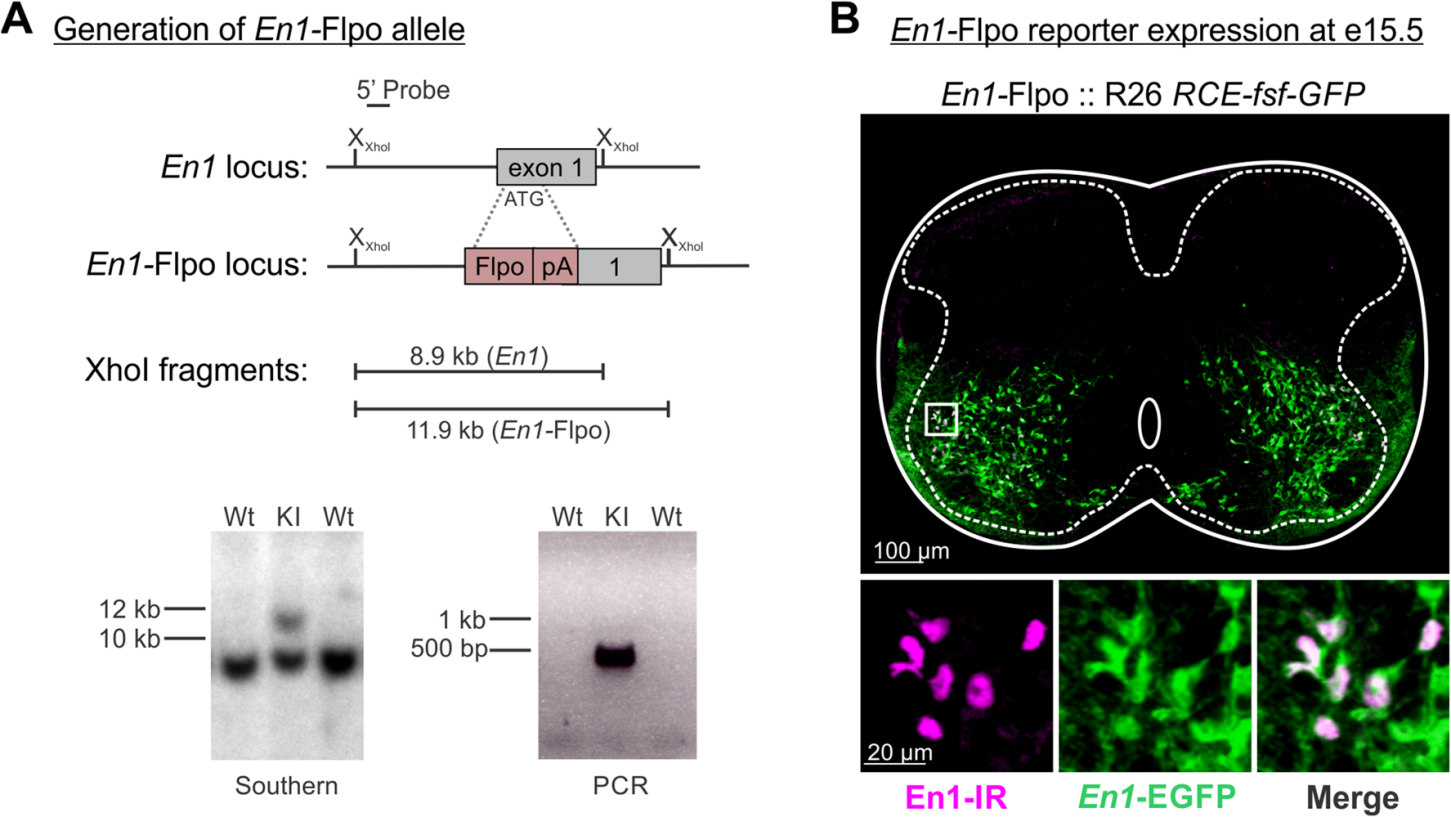
Generation of *En1*-Flpo mice. **(A)** Targeting strategy to generate *En1*-Flpo mice. Flpo was inserted into the ATG in the 1st exon of the *En1* genomic locus. Dotted lines represent approximate regions of homology in the targeting vector. Southern blot (bottom, left) of XhoI-digested genomic DNA with a 5’ probe external to the targeting vector identifies an 8.9 kb wild-type fragment and an 11.9 kb knock-in fragment. (Not shown: Deletion of selectable neomycin resistance gene flanked by loxP sites by crossing to *protamine*-Cre mice, which recombines the floxed PGK-Neo cassette in the male germline). PCR analysis (bottom, right) identifies a 484 bp band in the *En1::Flpo* allele. Southern and PCR blot show just two examples that were cropped form larger blots. The full lab hand-annotated blots are supplied at the end of the supplementary file. (**B)** En1 expression and reporter expression at e15.5. Top: low magnification confocal image stack of an e15.5 lumbar spinal cord section (20 µm thick) from a *En1*-Flpo :: R26 *RCE-fsf-GFP* animal, demonstrating GFP expression in ventral horn interneurons (green) and colocalization with En1 immunolabeling (magenta). Bottom: En1-IR neurons with GFP in the square region indicated in the low magnification image. Note that some lineage-labeled GFP + interneurons no longer express En1 protein at e15.5, likely reflecting rapid downregulation of En1 in many postmitotic V1 interneurons.

**Figure 5:**
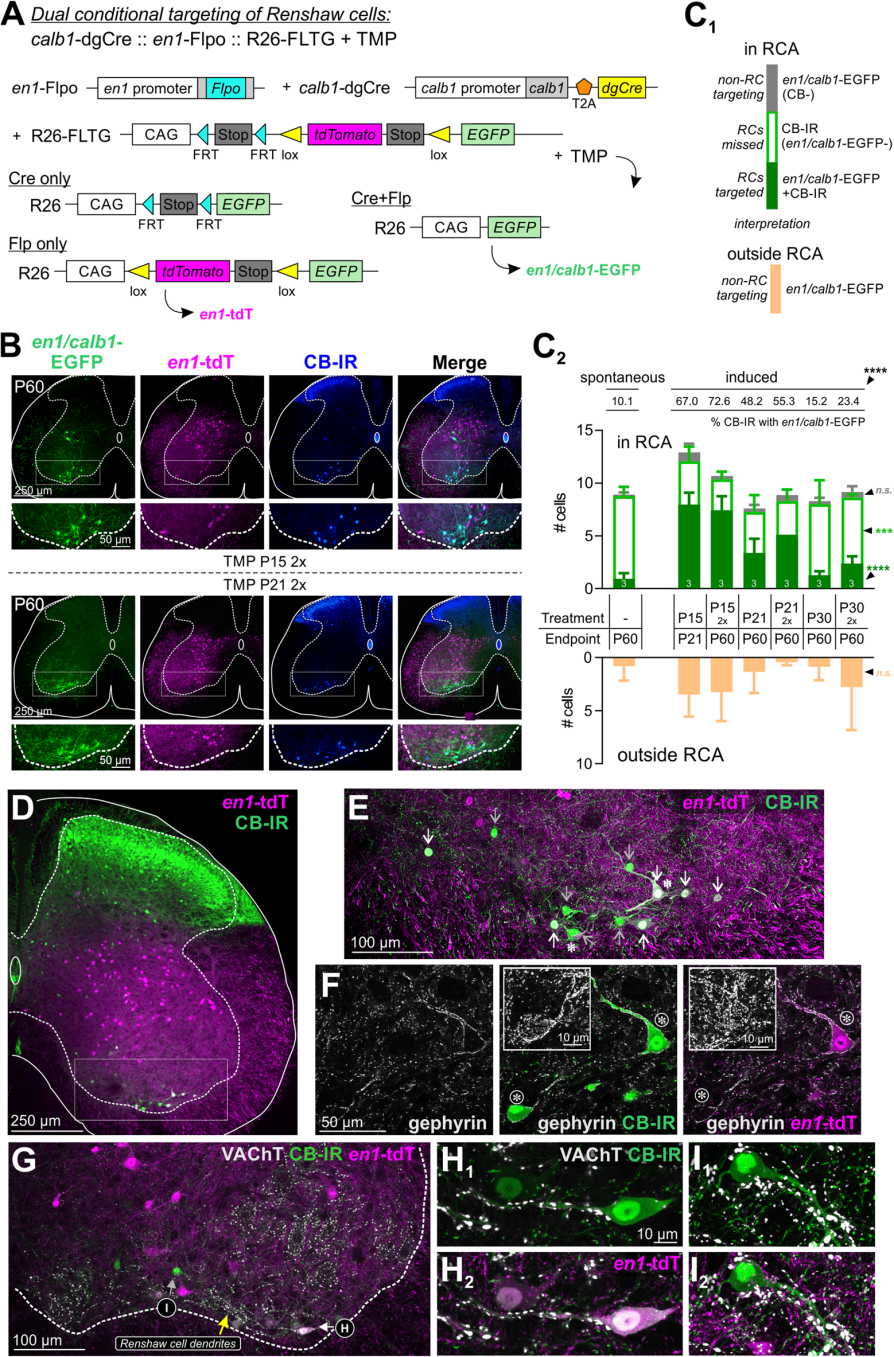
Genetic targeting of Renshaw cells by the intersection of *Calb1*-dgCre and *En1*-Flpo. **(A)** In the *Calb1*-dgCre :: *En1*-Flpo :: R26 FLTG system, embryonic expression of *En1* induces tdT in V1 interneurons. Cells expressing *Calb1* at the time of TMP administration remove tdT and upregulate EGFP. (**B)** Confocal image stacks showing genetic labeling at P60 following TMP injections at P15 or P21 (*En1/Calb1*-EGFP, green; *En1*-tdT, magenta; CB-IR, blue). Most RCs express EGFP but not tdT. V1 interneurons outside the RCA are more frequent after TMP at P15 compared to P21. **C.** Quantification of *En1/Calb1*-EGFP cells following TMP-induced or spontaneous dgCre recombination (no injection:—dash). The greatest RC labeling occurs after TMP at P15, with no difference according to age of analysis (P21 or P60). Dorsal non-RC V1 EGFP labeling is variable and not statistically different for any condition (Error bars = S.D.; asterisks in all graphs indicate significance level after One-Way ANOVAs across all conditions. *p < 0.05; **p < 0.01; ***p < 0.001; ****p < 0.0001, *n.s*. non-significant. For pair-wise comparisons and details of statistics and analysis, see Supplemental Tables 10–12 and Supplemental Fig. S6.) (**D–I).** *En1*-Flpo :: R26 FLTG labeling of RCs, defined by CB-IR and either (**E,F**) gephyrin or (**G–I**) cholinergic (VAChT) synapses (see “Methods”, CB-IR, green; *En1-*tdT, magenta; gephyrin or VAChT, white). (**D)** Low magnification confocal stack of CB-IR and *En1-*tdT labeling. (**E)** The deep ventral horn indicated in D shown at higher magnification; colored arrows point to RCs with tdT (white arrows) or without tdT (grey arrowheads). (**F)** Single confocal optical section containing two RCs with large bright gephyrin-IR clusters and CB-IR (asterisks in **E**,**F**). One expresses *En1*-tdT, the other does not. Insets show gephyrin clustering “en face” (superimposed 5–7 optical sections, 0.5 µm z-steps). (**G–I)** Medium magnification single optical section image (**G**), showing two RCs displayed at higher mag in (**H**,**I)**. VAChT contacts at high density on dendrites (**H**_**1**_,**I**_**1**_) in RCs with tdT (**H**_**2**_) or without tdT (**I**_**2**_) (five superimposed optical planes). Overall, 30% of RCs defined by CB-IR and their synaptic organization do not express tdT in *En1*-Flpo mice.

Compared to *Calb1*-dgCre alone, the *Calb1*-dgCre and *Pvalb*-Flpo intersection dramatically increases RC targeting specificity, but a significant number of dorsal horn cells were also labeled with this strategy. To avoid this dorsal interneuron population, we restricted *Calb1-*dgCre targeting to ventral V1 interneurons using a novel *En1-*Flpo animal (Fig. 4A). We first confirmed that V1 interneurons were correctly targeted in *En1*-Flpo mice using a Flp-dependent GFP reporter mouse (R26::*RCE-fsf-GFP*), resulting in GFP+ cells distributed in a manner characteristic of V1 interneurons (Fig. 4B). En1 immunoreactivity confirmed these cells were V1 interneurons, though not all GFP+ neurons were En1-IR due to rapid developmental downregulation of *En1* expression in many V1 interneurons. We then generated *Calb1-*dgCre *:: En1-*Flpo animals and crossed them to R26 FLTG reporter mice^26^ (see Fig. 5A). In this model, *En1*-Flpo labels V1 cells with tdT while TMP-timed *Calb1*-dgCre activity removes tdT and replaces it with EGFP in *Calb1*- expressing V1 cells (Fig. 5A,B).

We injected *Calb1-*dgCre *:: En1-*Flpo mice with either one or two doses of TMP at P15, P21, and P30 to avoid non-RC V1 interneurons expressing *Calb1* at earlier ages, followed by analysis at either P21 or P60. In contrast to the *Calb1*-dgCre :: *Pvalb*-Flpo model, there were no significant differences in the number or percentage of RCs targeted according to age of analysis or one or two TMP doses (Fig. 5C1, 5C2, Supplemental Fig. S6, Supplemental Tables 10, 11). The percentage of RCs decreased with TMP injection age, similar to all previous results with *Calb1*-dgCre. Two TMP doses at P30 labeled 24% of RCs at P60, whereas 73% and 57% of RCs were labeled when two doses of TMP were respectively injected at P15 or P21 (Supplemental Fig. S6; Supplemental Table 11). Notably, the *Calb1-*dgCre *:: En1-*Flpo model labeled fewer non-RCs compared to the *Calb1-*dgCre *:: Pvalb-*Flpo model, indicating an increased specificity of labeling. The number of *En1*/*Calb1*-EGFP non-RC V1 interneurons was negligible within the RCA, though we did observe a modest, somewhat variable number outside the RCA (averaging 0 to 7.4 cells per hemicord in different animals). The largest numbers of these cells were found after P15 TMP injections, but differences according to TMP injection dates were non-significant (Supplemental Fig. S6 and Supplemental Table 12). Most non-RC V1 interneurons were not CB-IR at the time of analysis (P60). The few adult V1 cells that expressed calbindin but are not RCs correspond to some sparse populations that were reported earlier^16,17^.

Despite improved specificity, targeting efficiency was lower than expected. To investigate this, we took advantage of the dual fluorescent reporters in the R26 FLTG mouse and pooled together all RCs labeled with either EGFP or tdT. Although the proportions of RCs labeled with EGFP or tdT varied according to TMP injection, unlabeled RCs remained relatively constant: 29.8% ± 8.0 (± S.D.) of RCs lacked any fluorescent protein across all experiments. This suggests incomplete RC targeting by the *En1-*Flpo allele (Supplemental Fig. S6; Supplemental Tables 10, 11). To confirm this, we generated one *En1-*Flpo R26 FLTG animal with all V1 interneurons labeled with tdT and observed that 33.8% of CB-IR cells in the RCA lacked tdT (Fig. 5D). Then we incorporated additional criteria for RC identification based on synaptic markers to exclude any influence of CB-IR non-RCs in the results. First, we identified RCs by CB-IR and the large gephyrin clusters on their cell bodies and proximal dendrites^27,28^ (Fig. 5E, 5F). Consistently, 32.4% of lumbar 4/5 large-gephyrin/CB-IR RCs lacked genetic labeling in *En1*-Flpo animals (n = 68 RCs). As further confirmation, we performed immunohistochemistry against the vesicular acetylcholine transporter (VAChT) to identify RCs by their distinctive high density cholinergic input on their dendrites^20,29,30^ (Fig. 5G): 27.4% of RCs such defined lacked tdT (n = 53 RCs; Figs. 5H_1_-H_2_ and 5I_1_-I_2_). We conclude that around 30% of RCs do not undergo Flp recombination in *En1-*Flpo animals. This differs from *En1-*Cre animals, in which 100% of RCs undergo genetic recombination^18^ (Fig. 1C). Therefore, *Calb1-*dgCre :: *En1-*Flpo mice display higher specificity but lower efficiency of RC targeting compared to the *Calb1-*dgCre :: *Pvalb-*Flpo model.

### Brain cells targeted in the intersection of *Calb1-*dgCre with either *Pvalb-*Flpo or *En1-*Flpo

**Figure 6:**
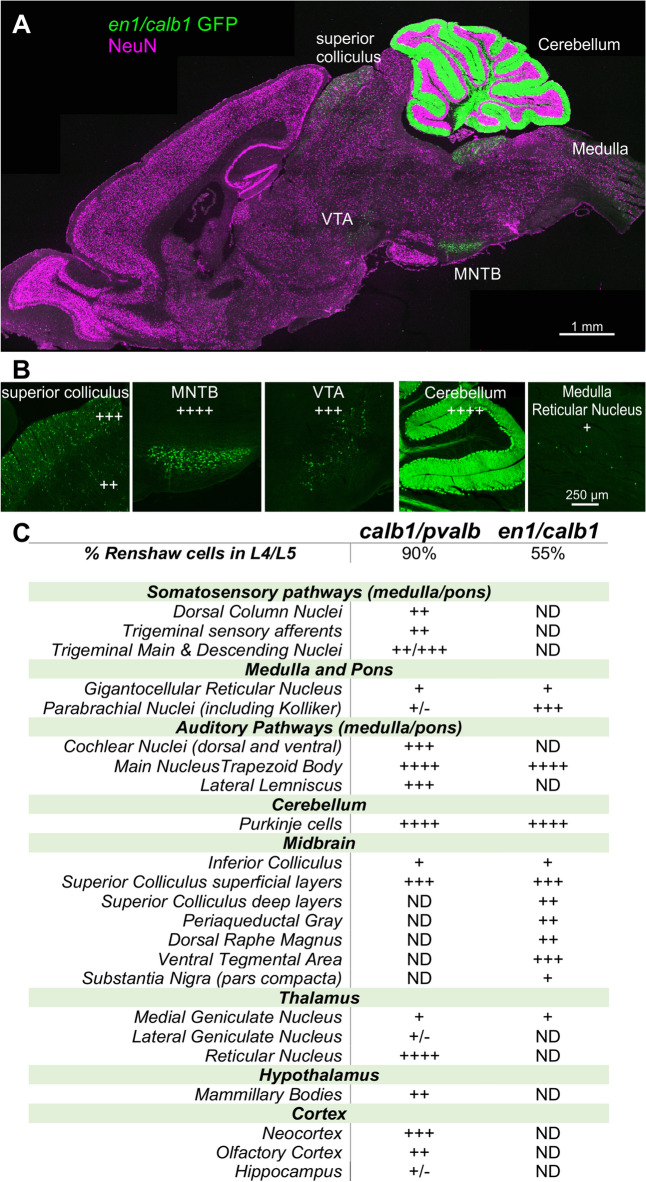
Distribution of brain cells targeted by *Calb1/Pvalb* and *En1/Calb1* intersections. **(A**) Genetic labeling in the brain of a *Calb1*-dgCre :: *En1*-Flpo :: R26 FLTG animal following two TMP injections at P21. Low magnification tiled single optical plane confocal image shows *En1/Calb1*-EGFP neurons (green) and NeuN-IR (magenta) for anatomical localization (En1-*tdT* not shown). Regions with high densities of labeled cells include Purkinje cells, the main nucleus of the trapezoid body (MNTB), the superior colliculus, the ventral tegmental area (VTA), and the parabrachial region (not visible in this section). (**B)** Higher magnification images of selected regions showing qualitative assessments of cell density (all are single optical sections). +  +  +  + , all neurons labeled; +  +  + high density; +  + medium density: + low density; ND non-detected. (**C)** Comparison of brain nuclei containing neurons labeled by the *En1/Calb1* intersection with 70% RC targeting efficiency and the *Calb1/Pvalb* intersection (R26 *RCE:dual-EGFP* reporter, TMP twice at P10) with 90% targeting efficiency.

To determine the extent to which these genetic strategies also label neuronal populations in the brain, we performed an analysis of the distribution of lineage-traced neurons in the brains of *Calb1*-dgCre/*Pvalb*-Flpo or *Calb1*-dgCre/*En1*-Flpo mice. Neurons in several brain areas were targeted by both intersections (Fig. 6A,B). We compared the brains of four *Calb1/Pvalb* animals at P60, two with 30% RC labeling following a single dose of TMP at P15, and two with 90% RC labeling following two doses of TMP at P10. Regardless of TMP dose, the same brain regions were genetically targeted, although sometimes at different labeling densities varying in the number of cells. We also analyzed the brains of two *Calb1-*dgCre *:: En1-Flpo* animals injected with two doses of TMP at P21 (55% RC labeling). In both *Calb1/Pvalb* and *En1/Calb1* brains, every cerebellar Purkinje cell was similarly labeled (Fig. 6). Both models also labeled all neurons in the main nucleus of the trapezoid body (MNTB) and many neurons in the superficial layers of the superior colliculus. Labeling in other brain regions differ between both models (Fig. 6C). Broadly speaking, *Calb1/Pvalb* neurons can be found throughout the brainstem, midbrain and forebrain, while *En1/Calb1* cells are focused to the midbrain.

### Spatially restricted targeting of RCs via intraspinal AAV injection

**Figure 7:**
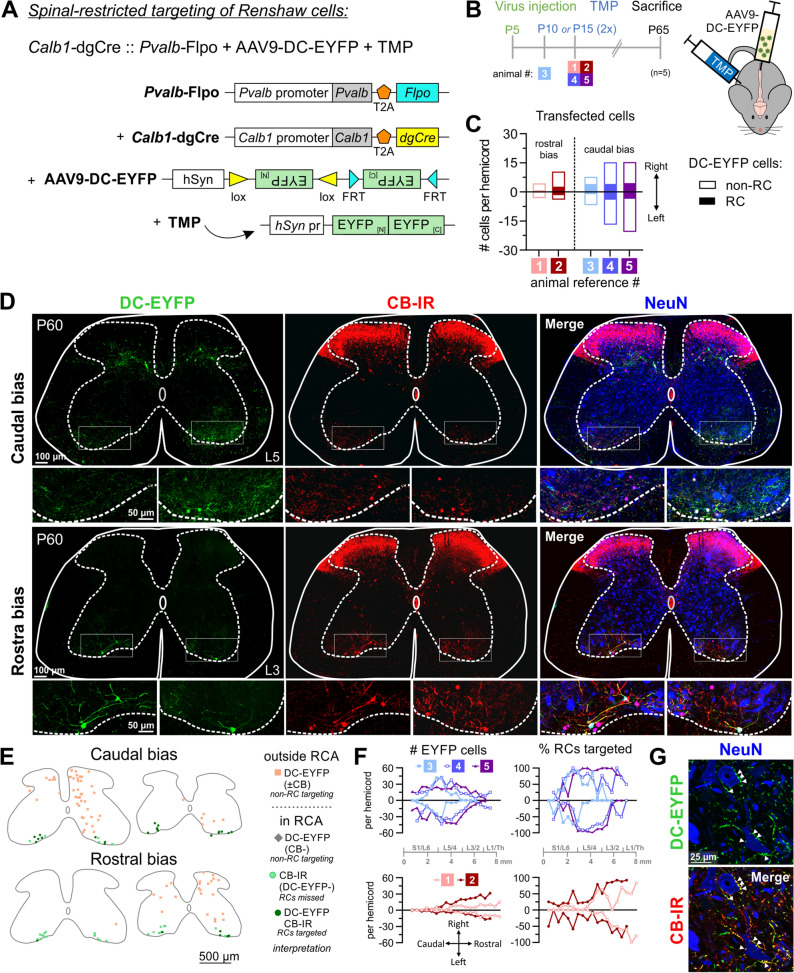
Spinal-restricted targeting of Renshaw cells in *Calb1*-dgCre :: *Pvalb*-Flpo animals using a dual-conditional AAV9 virus. (**A**) Schematic of the Calb1-dgCre :: Pvalb-Flpo + AAV9-DC-EYFP genetic system. For genetic targeting to occur, cells must be transfected with the virus, express synapsin, and undergo both Cre and Flp-recombination by expressing calbindin at the time of TMP administration and parvalbumin prior to age of sacrifice. This will invert the two EYFP sequences into the appropriate orientation and remove the recombination sites, enabling DC-EYFP expression. (**B)** Experimental paradigm. Five animals were sacrificed at P65 after receiving intraspinal injections of the AAV9-DC-EYFP virus between P5-P8 and two subsequent days of TMP administration at P10 or P15. (**C)** Quantification of transfected cells for each animal from upper sacral to lower thoracic spinal cord, presented as the average number of cells per hemicord (calculated by dividing the total number of DC-EYFP cells across all sections by the number of sections). Filled bars indicate Renshaw cells (DC-EYFP CB-IR cells in the RCA), and white bars indicate non-RCs. (**D–G)** Successful intersectional targeting of Renshaw cells in animals in which viral transfection (i.e. peak region and pattern of transfected cells) was biased caudally (top) or rostrally (bottom). (**D)** Confocal images show labeling in L5 or L3 segments (50 μm thick confocal stacks, 1 μm z steps: DC-EYFP, green; CB-IR, red; NeuN-IR, blue). (**E)** Representative cell plots in L5 and L3 demonstrating labeling after injections with a caudal or rostral bias. (**F)** Quantification of viral targeting along the rostrocaudal axis presented as total number of DC-EYFP cells (left) and percent of Renshaw cells targeted (total CB-IR cells in RCA expressing DC-EYFP; right). Each dot is the average of two serial sections. (**G)** Targeted interneurons synapse on motoneurons (single optical plane high magnification confocal image: DC-EYFP, green; CB-IR, red; NeuN-IR, blue). Arrows indicate dual-labeled EYFP + and CB-IR synapses on motoneurons.

To restrict genetic manipulations to the spinal cord while avoiding neurons in the brain, we tested the efficiency of targeting RCs with dual conditional AAV9 vectors injected into the postnatal spinal cord. For this purpose we used only *Calb1-*dgCre :: *Pvalb-*Flpo animals because RCs in these mice maintain Cre and Flpo expression postnatally, whereas *En1* is downregulated in RCs embryonically and appears to not maintain expression of recombinases at postnatal ages. We injected five animals (ages P5-P8) with AAV9 carrying a dual conditional *eyfp* gene under the control of the *hSyn* promoter (Fig. 7A)^31^, targeting the dorsal midline with a rostral bias (upper lumbar injection, n = 2) or caudal bias (caudal lumbar injection, n = 3), followed by administration of TMP twice at P10 or P15 (Fig. 7B,C; see “Methods”). Serial sections were aligned according to cytoarchitectonic landmarks and quantified. All animals displayed EYFP bilaterally in both the dorsal and ventral horns (Fig. 7C-F), indicating adequate penetrance of AAV9 throughout the dorso-ventral extent of the spinal cord. The total number of cells labeled varied across animals, with approximately 3–25 EYFP + cells per hemicord from S1-T13 (Fig. 7C). In the L4/L5 region, this number increased in caudal bias animals (15.0–33.8 cells per hemicord) but not rostral bias animals (0.7–9.0 cells per hemicord) (Fig. 7F, left). While 14.7–34.7% of RCs throughout the lumbar region were EYFP labeled overall, the percentages increased depending on the lumbar segments analyzed and the location of the injection. For example, in L4/L5, 33.6–81.2% of RCs were labeled in caudal bias animals and only 1.7–30.9% in rostral bias animals (Fig. 7F, right). The high degree of inter-animal variability indicates that the rostro-caudal spread of AAV9 needs to be confirmed in each animal, but in all cases the majority of RCs were targeted around the injection site: 83.3—100% of RCs in L4/5 or L1/L2 segments after caudal or rostral bias injections, respectively. EYFP labeling of RCs included the cell body, dendrites, axons and synapses on motoneurons (Fig. 7G), suggesting its utility for examining synaptic connectivity. Together, the results show that intraspinal AAV9 transduction in *Calb1*-dgCre :: *Pvalb*-Flpo animals targets a large percentage of RCs in a region comprising approximately 2 segments above and below the injection site.

## Discussion

We have described two strategies for genetically targeting RCs, including the optimal timing and dosage of TMP for inducing Cre activity from *Calb1*-dgCre alleles. *Calb1*-dgCre combined with *Pvalb*-Flpo targeted 90% of RCs and 70% of RCs when combined with *En1*-Flpo. Each model has unique properties. Genetic targeting of RCs continues to increase postnatally after TMP injection in the *Calb1/Pvalb* model due to progressive upregulation of *Pvalb* during late maturation. In contrast, RC targeting in the *Calb1/En1* model is expected to occur within 36 h after TMP and not to change at later times after TMP injection. Therefore, the *Calb1/En1* model is better suited for experiments requiring accurate timing, while the *Calb1/Pvalb* model increases coverage to almost the full RC population through and extended period of time. Both result in additional targeting of non-RC spinal interneurons, as well as neurons in the brain, quite prominently Purkinje cells. This limits their utility for whole-animal in vivo experiments using transgenic mice to introduce dual-conditional activity modifiers (optogenetics, DREADDs) or cellular ablation (diphteria toxin), as supraspinal systems will also be targeted. One alternative approach is to introduce dual Cre and Flp dependent transgenes locally in the spinal cord via viral delivery as demonstrated in our study. For this purpose, the *Calb1/Pvalb* model needs to be used because it retains Cre and Flp expression in postnatal RCs. Neither model, however, results in complete specificity within the spinal cord. Confounds introduced by a few dorsal horn interneurons in the *Calb1/Pvalb* model or a few other V1 interneurons in the *Calb1/En1* model will need to be tested in each experiment. The non-RC spinal neurons labeled in each model are different. Therefore, increased specificity can be predicted in a triple intersection using novel commercially available *En1*-Dreo^32^ or *Pvalb*-Dreo^24^ mice combined respectively with the *Calb1*-dgCre/*Pvalb*-Flpo or *Calb1*-dgCre/*En1*-Flpo models. In particular, because the *En1-*Dreo relies on a 2A-Dre transgene that does not disrupt endogenous expression of *En1*, this model has the further advantage of maintaining *En1* expression. This could be relevant in some studies since *En1* gene heterozygosis has been shown to accelerate aging deficits in some neurons ^33,34^. Activity modifier genes dependent on combinations of Cre or Flp with Dreo are not yet available, but this will likely change in the future.

We were surprised to find that *En1*-Flpo is less effective than *En1*-Cre targeting RCs, although both are constructed with similar insertions in the ATG of exon1 (see^18^). Originally, Cre was suggested to be more effective than Flp in mammalian cells because of differences in thermostability^35^, leading to development of “enhanced” Flpe versions^36^. We used “optimized” Flpo which was shown to have even higher recombination efficacy than Flpe^37^. In RCs, however, En1-Flpo was less effective than En1-Cre, perhaps because RCs are the first En1 cells to be generated from the spinal cord p1 domain and downregulate En1 in early embryo. Thus, one possibility is that Cre or Flpo expression dependent on the En1 promoter might be short lived in the last-generated RCs, limiting recombinase activity and uncovering differences between Cre and Flpo not evident with longer or higher recombinase expression. RCs not targeted in *En1*-Flpo mice did not display differences based on location or synaptic features. Future studies should more systemically study whether non-targeted RCs represent a unique subpopulation.

Across all models using *Calb1*-dgCre to target RCs, two features of this allele need careful consideration. First, there was considerable leakiness in the system, though this is not apparent in RCs in the first three postnatal weeks. The fact that increased spontaneous recombination in RCs is only observed after the first month and it is marginal until 6 months of age, suggests that it should not interfere with studies that aim to target RCs in young adults for anatomical or neurophysiological studies. Fortunately, genetic intersection with *Pvalb* or *En1* dramatically diminishes the number of cells targeted through TMP-independent dgCre activity.

Second, RC targeting efficiency changes with age of TMP injection. When considering the reduced RC targeting after TMP administration at older ages, it is possible that the *Calb1* promoter becomes less active in older RCs despite CB-IR remaining detectable. Alternatively, proteosome degradation of dgCre might become more efficient in mature RCs. Whether any of these mechanisms can be overcome by more/higher doses of TMP will need to be tested. Another possibility is to set *Calb1*-dgCre in homozygosis (the allele retains endogenous Calb1 expression), but it is predictable that higher dgCre expression would increase spontaneous recombination rates and reduce timing accuracy. It is also unclear from a mechanistic point of view why when using *Calb1/Pvalb* intersectional approaches, TMP injected in the first postnatal week induces recombination in fewer RCs compared to *Calb1*-dgCre alone and dual injections of TMP were necessary at any age to maximize RC targeting. In contrast, a double TMP dose did not increase targeting efficiency in the *Calb1/En1* intersection. Despite the lack of clear explanations for these observations, double TMP doses were empirically found to increase Cre recombination when this was weak and have no deleterious actions when this was maximized. Thus, in the future we will follow dual injection protocols when using *Calb1*-dgCre alleles in intersectional paradigms.

In summary for the two models proposed second postnatal week TMP injections induces efficient Cre recombination in RCs with relatively high specificity and avoids compensatory changes due to genetic targeting during earlier development. Future studies using these models should focus in relatively young adults to prevent accumulation of off-target cells due to TMP-independent dgCre recombination events.

The models we describe here overcome the limitations reported for BAC *Chrna2*-Cre mice^14,15^. BAC *Chrna2*-Cre mice are similarly efficient at targeting 86–94% of RCs in the RCA; however, Cre recombination occurs through embryonic development, which can confound functional studies. Moreover, we systematically analyzed targeting of non-RCs across the brain and spinal cord. This level of characterization has not yet been reported for this BAC *Chrna2*-Cre line with high levels of RC targeting, despite the fact that some dorsal horn neurons^15^ and many neurons in the brain^13^ express *Chrna2*a. The exact spinal and brain cells targeted need to be fully mapped for each mouse BAC line because expression in BAC transgenics frequently differs from endogenous gene expression depending on the specifics of gene regulatory sequences and cell gene controls incorporated into the BAC^38^. The published BAC *Chrna2*-Cre line with high levels of RC targeting is not available.

## Conclusions

We characterized two models for genetic targeting of RCs using currently available mouse lines that are widely available to the research community. Each has specific advantages or limitations that suit them to different types of experiments, enabling the targeting of almost all RCs, or alternatively, providing a way to sparsely label RCs or target RCs in defined spinal segments. Because labeling includes cell bodies, dendrites, axons and synapses, these approaches will be particularly useful in anatomical investigations of RC connectivity, facilitating analyses of RC output at the population or single-cell level. This information is critical to further refine computational models of RC function. Moreover, the viral strategies employed here can be used with optogenetic, chemogenetic or cellular ablation methodologies for temporal and spatial control of RC activity in selected spinal cord segments. Taken together, these models provide improved genetic access to RCs to facilitate future studies aimed at testing the functional role of RC circuitry in the spinal cord motor network.

## Methods

### Animals

All experiments and procedures were performed according to NIH guidelines and approved by the Institutional Animal Care and Use Committee of Emory University and. Animals were maintained on a C57BL/6 background. Tail and toe tips from neonatal mice were routinely collected for PCR genotyping. All transgenic lines used in this study can be found in Table 1. *En1*-Cre, *En1*-Flpo, and *Mafb-GFP* mice were maintained in heterozygosis. All other lines were maintained in homozygosis. Various breeding schemes were used to generate single-conditional (Cre) and dual-conditional (Cre and Flp) experimental animals of various types. Animals were genotyped by in-house PCR or by Transnetyx with real-time PCR using primers listed in Supplemental Table 13.

**Table 1:** Transgenic mice use in this study.

Name	Brief description & source
***En1-*Cre**; *En1 tm2(cre)Wrst/J; Cre*	Cre inserted into ATG of 2nd exon^18^. Donating invetsigator Dr. Martyn Goulding, Salk Institute
***Mafb-*GFP**	EGFP inserted in MafB gene^23^^.^ Donating invetsigator Dr. Satoru Takashi, Tsukuba University
***Calb1-*dgCre**; *B6.Cg-Gt(ROSA)26Sortm9(CAG-tdTomato)Hze/J*	Viral 2A oligopeptide and destabilized EGFP/Cre fusion gene (dgCre) inserted downstream of Calb1 stop codon^39^ (JAX#023531). Donating investigator Hongkui Zeng, Allen Institute for Brain Science
***Pvalb-*Flpo**; *Pvalb-2A-FlpO-D*	Viral 2A oligopeptide and Flp inserted downstream of PV stop codon^24^ (JAX#022730). Donating investigator Dr. Hongkui Zeng, Allen Institute for Brain Science
***En1-*Flpo**	Flpo was inserted into the ATG in the 1st exon
**Ai9 R26 *lsl-tdT***; *B6.Cg-Gt(ROSA)26Sortm9(CAG-tdTomato)Hze/J)*	lox-flanked CAG-tdTomato-WRE Cre reporter targeted to the R26 locus^40^ (JAX#007909). Donating investigator Dr. Hongkui Zeng, Allen Institute for Brain Science
**R26 *RCE:dual-EGFP***; *Gt(ROSA)26Sortm1(CAG-EGFP)Fsh/Mmjax*	lox- and FRT-flanked CAG-EGFP dual Cre/Flp reporter targeted to the R26 locus^41^ (MMRRC#032036-JAX) Donating investigator Dr. Gord Fishell, Smilow Research Center, New York University
**R26 FLTG**; *B6.Cg-Gt(ROSA)26Sortm1.3(CAG-tdTomato,-EGFP)Pjen/J*	frt-flanked STOP and loxP-flanked tdTomato::STOP dual Cre/Flp reporter upstream of the EGFP targeted to the R26 locus^42^ (JAX#026932) Donating investigator Dr. Patricia Jensen, National Institute of Environmental Health Sciences
**R26 *RCE-fsf-GFP***	Flp-dependent eGFP reporter under control of the CAG promoter at the R26 locus^44^ (#32038-JAX) Donating investigator Dr. Gord Fishell, Smilow Research Center, New York University

*En1::Flpo* mice were generated as described^45^. Briefly, Flpo, a codon-optimized version of Flp recombinase^46^ was inserted into the ATG in the 1st exon of the *En1* genomic locus, generating a null allele that simultaneously enables lineage tracing. Positive ES cell clones were screened by Southern blot analysis and microinjected into blastocysts, and the resulting chimeric mice were crossed to C57BL/6J females. The neomycin selectable cassette was removed using *Protamine::Cre* mice (JAX #003328). Mouse strains were maintained on a C57BL/6J background, and were backcrossed for > 6 generations.

### Trimethoprim (TMP) administration

Trimethoprim (TMP; Sigma T7883) was reconstituted in DMSO to 100 mg/mL and diluted with saline to produce 5 or 10 mg/mL (25% DMSO) or 12.5 mg/mL solutions (35% DMSO) (prepared same day). Animals were injected intraperitoneally (50, 100, or 125 mg/kg) with a single dose or with two doses separated by 24–48 h.

### Virus and surgeries

The Emory Viral Vector Core prepared an AAV9 carrying the pAAV-hSyn Con/Fon EYFP plasmid (Addgene #55650; Depositor: Karl Deisseroth)^31^. Five animals between P5-P8 were anesthetized with isoflurane until a surgical plane of anesthesia was achieved (induction: 4%; maintenance: 2%, both in 100% O_2_) and given a subcutaneous injection of 0.05 mg/kg buprenorphine to reduce postsurgical pain. A small skin incision was made in the dorsal surface below the last thoracic vertebrae. Using a glass micropipette, we slowly injected 0.5 μL of virus (2.6 × 10^13^ genomic copies/mL) in the gap in vertebrae between Th13 and L1 (n = 2, rostral bias) or L3 and L4 (n = 3, caudal bias). The skin was then aligned and sutured back together. Animals were monitored daily for the first week after surgery; none exhibited signs of pain or distress. These animals received two injections of TMP at P10 or P15.

### Tissue preparation

Animals were deeply anesthetized with Euthasol and perfused transcardially with 4% paraformaldehyde in 0.1 M phosphate buffer (pH 7.4). Spinal cords and brains were collected and post-fixed overnight in the same fixative. The tissues were stored in 30% sucrose in 0.1 M PB at 4 °C until use.

### Histological processing and immunohistochemistry

Lumbar segments 4 and 5 (L4/L5) were blocked and 50 µm thick freezing sliding microtome sections prepared. Sections from virus-injected animals were mounted serially and processed on slides; all other spinal cord sections were processed “free‐floating”. Full brains were sectioned longitudinally on a cryostat and processed on slides. The midbrain and hindbrain were blocked and sectioned coronally using a freezing sliding microtome and processed free-floating (50 µm thick). All sections were blocked with normal donkey serum diluted 1:10 in 0.01 M phosphate buffer saline (PBS) with 0.1% or 0.3% Triton-X-100 (PBST). Sections were then incubated at room temperature in different combinations of primary antibodies (Supplemental Table 14) diluted in PBST for one day. For calretinin and parvalbumin staining, sections were incubated for two days to improve antibody penetration in high salt PBST (2.5% NaCl) to reduce background. Immunoreactive sites were revealed with mixtures of species-specific anti-IgG secondary antibodies made in donkey (Jackson Immunoresearch, Supplemental Table 15) diluted 1:100 in PBST. Sections were thoroughly washed in PBS, mounted on glass slides if free-floating, and coverslipped with Vectashield anti-fading medium (Vector).

### Confocal imaging and image analysis

All images were acquired using an Olympus FV1000 confocal microscope. Spinal cords were imaged at low magnification (10× , N.A. 0.4, 1 μm z steps). Brains were imaged using the same objective (4 μm z steps). Motor neurons in virus-injected animals were imaged using a 60× objective with × 2 digital zoom (N.A. 1.35, oil-immersion, 0.5 μm z steps). Images were imported into the neuron tracing software Neurolucida for analysis (version 12.0, MicroBrightField).

#### RC area definition

A topographical method for identifying Renshaw cells based on calbindin expression was developed in order to consistently assess the proportion of Renshaw cells that were successfully targeted. The vertical distance between the central canal and the ventral-most border of the gray-white matter in lamina IX was measured, and the Renshaw cell area (RCA) was defined as the ventral 45% of this region based on calbindin-immunoreactivity (IR) and lineage labeling or expression of the transcription factors *En1* and *MafB*, respectively (Fig. 1C). The 45% limit was empirically obtained by confirming in spinal cord sections of different age inclusion of > 95% of Renshaw cells (RCs). The use of a percentage distance, rather than absolute distance, allowed us to apply the same criteria to spinal cords of different ages and with significantly different sizes. It needs to be emphasize that this criteria is valid for Lumbar 4 and 5 segments. In other segments the shape of the spinal cord varies and spatial criteria for the RCA will need to be validated in the future.

#### Cell counting

For each animal, approximately 12 ventral horns were analyzed (between 7 and 22; see Supplemental Tables). Different antibodies and analyses were applied depending on the experiment and animal genotype and are described in Supplemental Table 16. Briefly, the RCA was traced in Neurolucida as described above, and counts were acquired for cells expressing genetic reporters and/or calcium binding proteins (calbindin, calretinin, parvalbumin). Only cells within the RCA were counted in single-conditional *Calb1-*dgCre/ + animals, as we wished to quantify efficiency of RC targeting in these animals. All genetically targeted cells (both inside and outside the RCA) were counted in dual-conditional animals (*Calb1-*dgCre/ + :: *Pvalb-*Flpo/ + :: R26 *RCE:dual-EGFP/* + or *Calb1-*dgCre/ + :: *En1-*Flpo/ + :: R26 FLTG). The percent of various populations of cells that were co-localized with a genetic reporter or another marker were then calculated, as described in each figure legend and corresponding Supplemental Tables. For virus-injected animals, these same analyses were applied to serial sections, which were aligned across animals using NeuN-IR to confirm segment transitions according to cytoarchitectonic landmarks. Each point in the resulting plots (Fig. 7F) is the average of two adjacent sections.

#### Synaptic markers

To confirm lack of universal targeting of RCs in *En1*-Flpo animals, we combined CB-IR with either gephyrin or vesicular acetylcholine transporter (VAChT) immunolabeling to identify RCs based on location, calbindin expression, and synaptic characteristics. We used one *En1*-Flpo :: R26 Ai9 * lsl-tdTomato* animal and analyzed all identified RCs in L4/5 segments through 6 hemicords (68 RCs) in the calbindin/gephyrin combination and 15 hemicords (73 RCs) in the calbindin/VAChT combinations. The percentage of identified RCs expressing tdTomato was then calculated. RCs were defined as CB-IR cells with large gephyrin clusters in the cell body and proximal dendrites, or CB-IR cells with a high density of large VAChT-IR contacts on their dendrites. More hemicords were used to analyze similar numbers of RCs based on VAChT immunolabeling because the higher difficulty of sampling RCs with long dendrites in 50 µm thick sections.

#### Figure composition

Figures were composed using CorelDraw. Pseudocolors were chosen from lookup tables in Fluoview or ImageJ. Image brightness and contrast were optimized with Image Pro Plus or ImageJ. Some images were sharpened using either a “sharpen” or “high-gauss” filter. All manipulations were done on the entire image. Digital manipulations were minimal and did not alter information content in the images.

### Statistics

For each condition, we averaged data from approximately 3 to 5 animals (between 2 and 12 animals across all experiments). “n” usually refers to number of animals (except when indicated in “Results”), and inter-animal variability was kept low by performing repetitive measurements in each animal before obtaining one average per animal. The exact details can be found in corresponding Supplemental Tables and in the Results and preceding sections detailing each of the analyses. We used one-way ANOVAs to reveal significant differences according to different experimental conditions. If we observed significant differences, we used Bonferroni post hoc t-tests for pairwise comparisons. All α values were set at 0.05. Sample sizes were set to power = 0.80 and varied according to sample variance and the size of the effect. If effect sizes were too small (10% difference), we did not seek incrementing sample sizes to increase power but interpreted any change too small to be of relevance.

### Ethics verification

This study was conducted and reported in accordance with ARRIVE guidelines. The institutional and licensing committees approving the experiments are identified at the beginning of the Materials and Methods. All animal experiments were conducted in accordance with relevant guidelines and regulations. All experimentation and data analyses were performed in conformity with ARRIVE guidelines.

## Supplementary Information

Supplementary Information 1.

Supplementary Information 2.

## Data Availability

All data generated or analyzed during this study are included in this published article and its Supplementary Information files.
