# Supplementary material for: Genetic targeting of adult Renshaw cells using a Calbindin 1 destabilized Cre allele for intersection with Parvalbumin or Engrailed1
Source: Sci Rep. 2021 Oct 6;11:19861. doi: 10.1038/s41598-021-99333-6 (PMC8494874; doi:10.1038/s41598-021-99333-6)
Supplement: Supplementary file 1 — Supplementary Information 1. [file 41598_2021_99333_MOESM1_ESM.pdf]

Supplemental Materials

Genetic targeting of adult Renshaw cells using a *calbindin1* destabilized Cre allele for intersection with *parvalbumin* or *engrailed1*

Alicia R. Lane[1], Indeara C. Codgell[1], Thomas M Jessell[3], Jay B. Bikoff[2], & Francisco J. Alvarez[1]*

[1]Department of Physiology, Emory University, Atlanta, GA 30322

[2]Department of Developmental Neurobiology, St. Jude Children's Research Hospital, Memphis, TN 38105, USA

[3]Department of Biochemistry and Molecular Biophysics, Columbia University, New York, NY 10032, USA

*** Corresponding Author:** francisco.j.alvarez@emory.edu

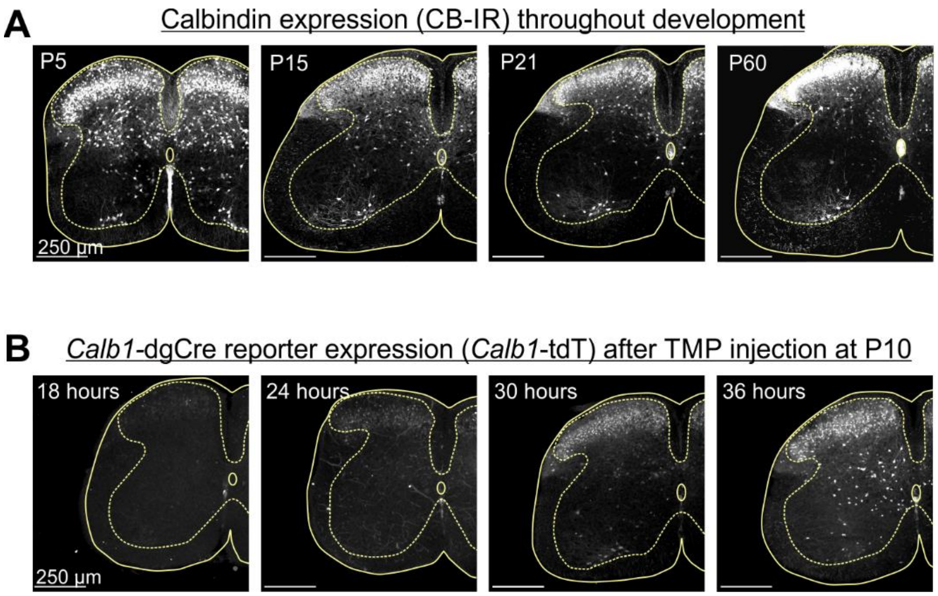

Supplemental Figure S1.

(A) Downregulation of calbindin expression with age. Calbindin-immunoreactivity (CB-IR) is greatest in the spinal cord early in development. From P5 to P60, CB-IR disappears from many spinal interneurons but is selectively maintained in dorsal horn superficial laminae neurons and in Renshaw cells in the most ventral region of the spinal cord.

(B) Timing of tdTomato (tdT) expression in *Calb1*-dgCre :: Ai9 R26 *lsl-tdT* animals after trimethoprim (TMP) administration. Each panel depicts *Calb1*-tdT labeling without amplification by immunolabeling after TMP injection at P10. 18 hours after TMP administration, only a few very weakly labeled cells are visible. Over time, the intensity of *Calb1*-tdT expression and the number of cells labeled increases. At 30 hours, superficial laminae cells and Renshaw cells were weakly labeled. By 36 hours after injection, many spinal interneurons in all laminae were strongly labeled. Labeling was similar at 36 and 42 hours (not shown). Thus, a minimum of approximately 36 hours after TMP administration is required to observe peak reporter expression.

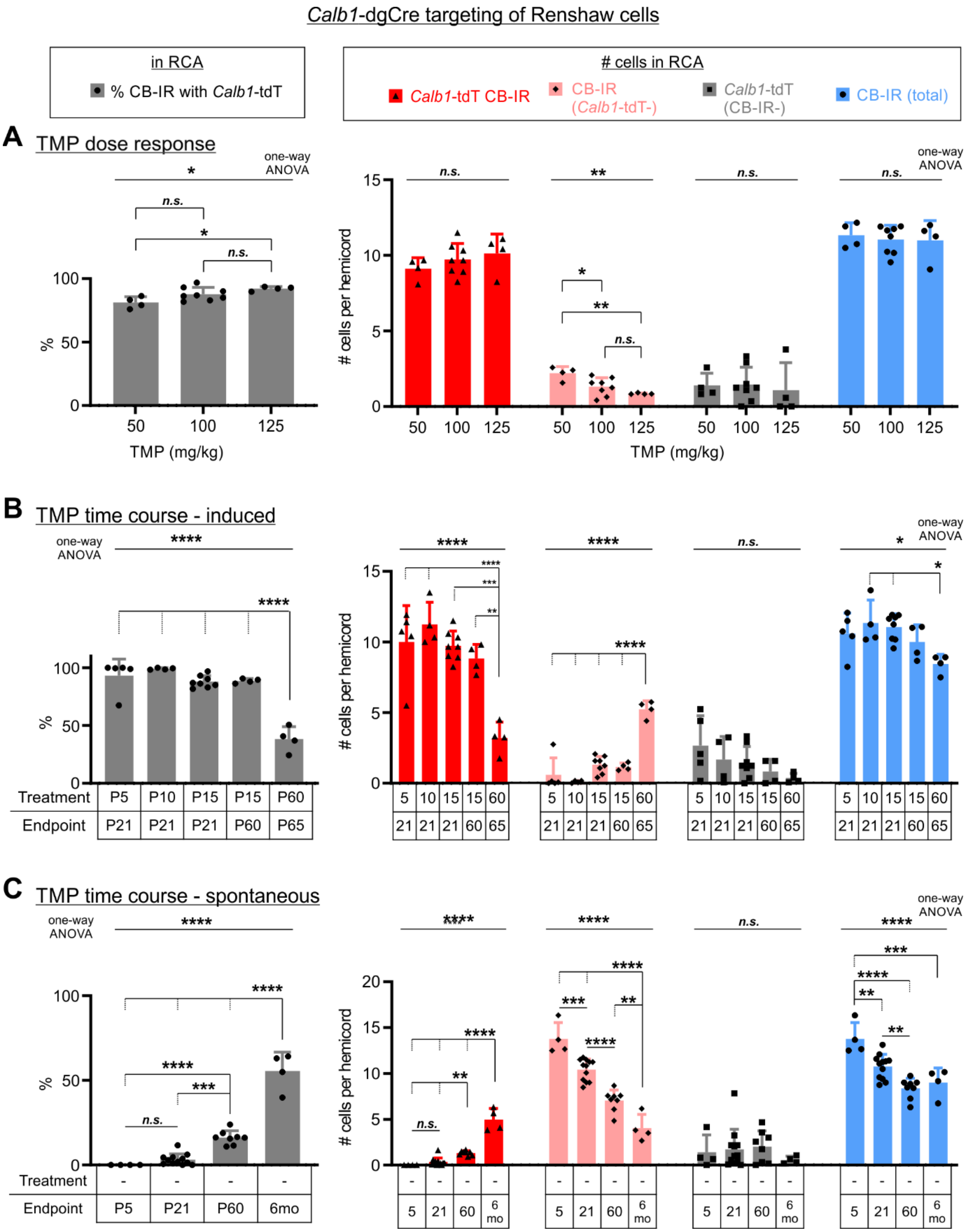
Calb1-dgCre targeting of Renshaw cells
in RCA
% CB-IR with Calb1-tdT
# cells in RCA
Calb1-tdT CB-IR
CB-IR (Calb1-tdT-)
Calb1-tdT (CB-IR-)
CB-IR (total)
A
TMP dose response
one-way ANOVA
n.s.
%
cells per hemicord
TMP (mg/kg)
B
TMP time course - induced
Treatment
Endpoint
P5
P10
P15
P60
P21
P65
C
TMP time course - spontaneous
6mo
6 mo

Supplemental Figure S2.

Genetic *Calb1*-tdT labeling and calbindin expression according to TMP dose (A), TMP paradigm (B), and in the absence of TMP (C) in *Calb1*-dgCre :: Ai9 R26 *lsl-tdT* animals. In all cases, data is presented as percentages of CB-IR cells in the RCA (left; gray) or number of cells per RCA labeled with different combinations of *Calb1*-tdT and CB-IR (right). Blue bars indicate the average total number of CB-IR cells per RCA analyzed in each experiment. These are consistent when animal age is maintained constant (dose analysis) but decreases with age, as expected, due to neuropil expansion and reduced neuronal density. Each data point represents one animal. Statistical details (one-way ANOVAs followed by Bonferroni corrected multiple pair comparisons) are described in accompanying Supplemental Tables 2-5.

(**A**) Increasing TMP dose above 100 mg/kg does not affect *Calb1*-tdT labeling (see Supplemental Table 3).

(**B**) The efficiency by which TMP induces *Calb1*-tdT labeling of RCs is high from P5 to P15 and is far reduced at P60 (see Supplemental Table 4).

(**C**) Spontaneous recombination (*Calb1*-tdT labeling in the absence of TMP) increases over time and occurs in over 50% of CB-IR cells in the RCA by 6 months of age (see Supplemental Table 5).

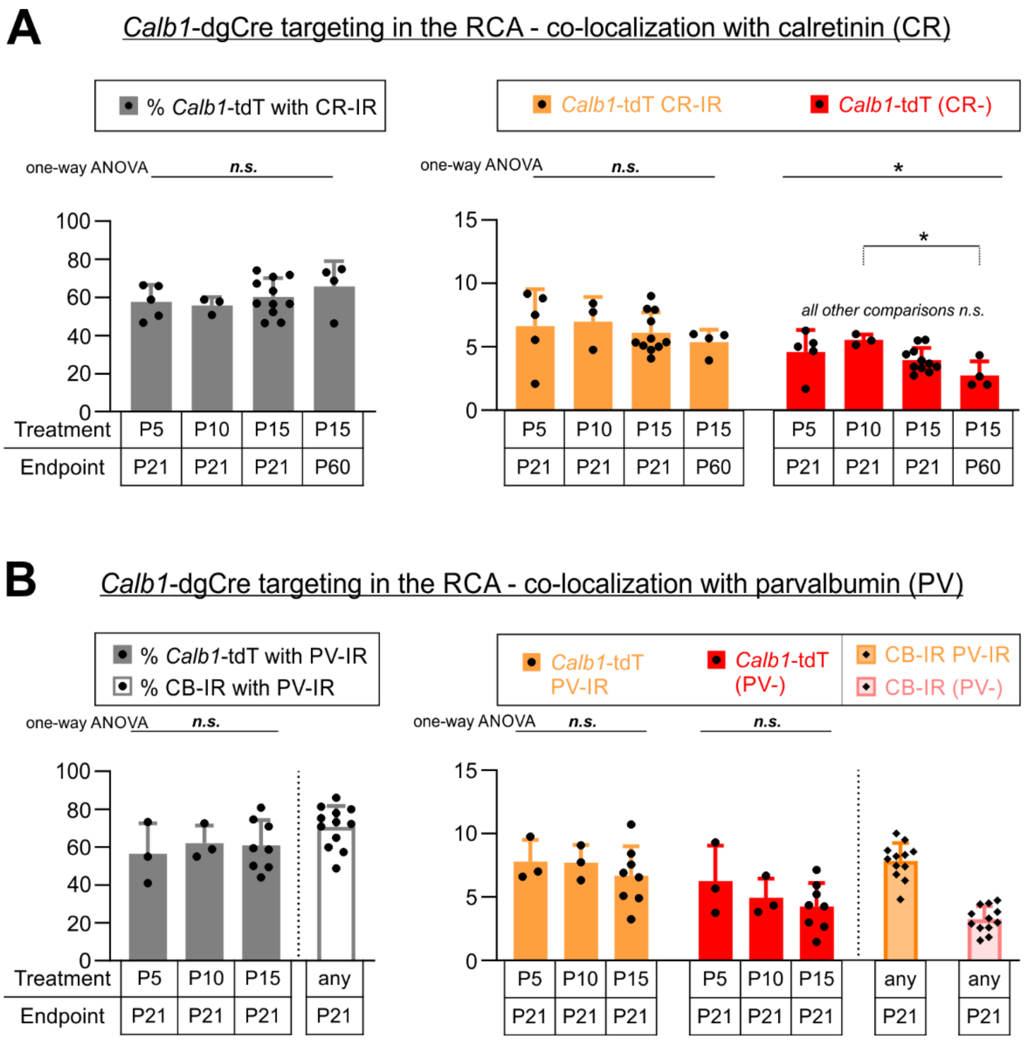

Supplemental Figure S3.

Numbers and percentages of *Calb1*-tdT cells in the RCA expressing calretinin (CR, panel A) or parvalbumin (PV, panel B) in *Calb1*-dgCre :: Ai9 R26 *lsl-tdT* animals. Data are presented as percentages of *Calb1*-tdT cells in the RCA immunolabeled with CR or PV (left) and number of *Calb1*-tdT cells per RCA with or without CR-IR or PV-IR (right). Each data point represents one animal. Statistical details (one-way ANOVAs followed by Bonferroni corrected multiple pair comparisons) are shown in Supplemental Table 6.

(**A**) When TMP is administered from P5 to P15, similar numbers and proportions of *Calb1*-tdT cells in the RCA express CR independent of TMP administration time or age of analysis.

(**B**) When TMP is administered from P5 to P15, similar numbers and proportions of *Calb1*-tdT cells in the RCA express PV independent of TMP administration time or age of analysis. *Calb1*-tdT cells expressing CR and PV are similar in proportion and number. The number and proportion of *Calb1*-tdT cells with PV-IR is similar to that of CB-IR cells with PV-IR.

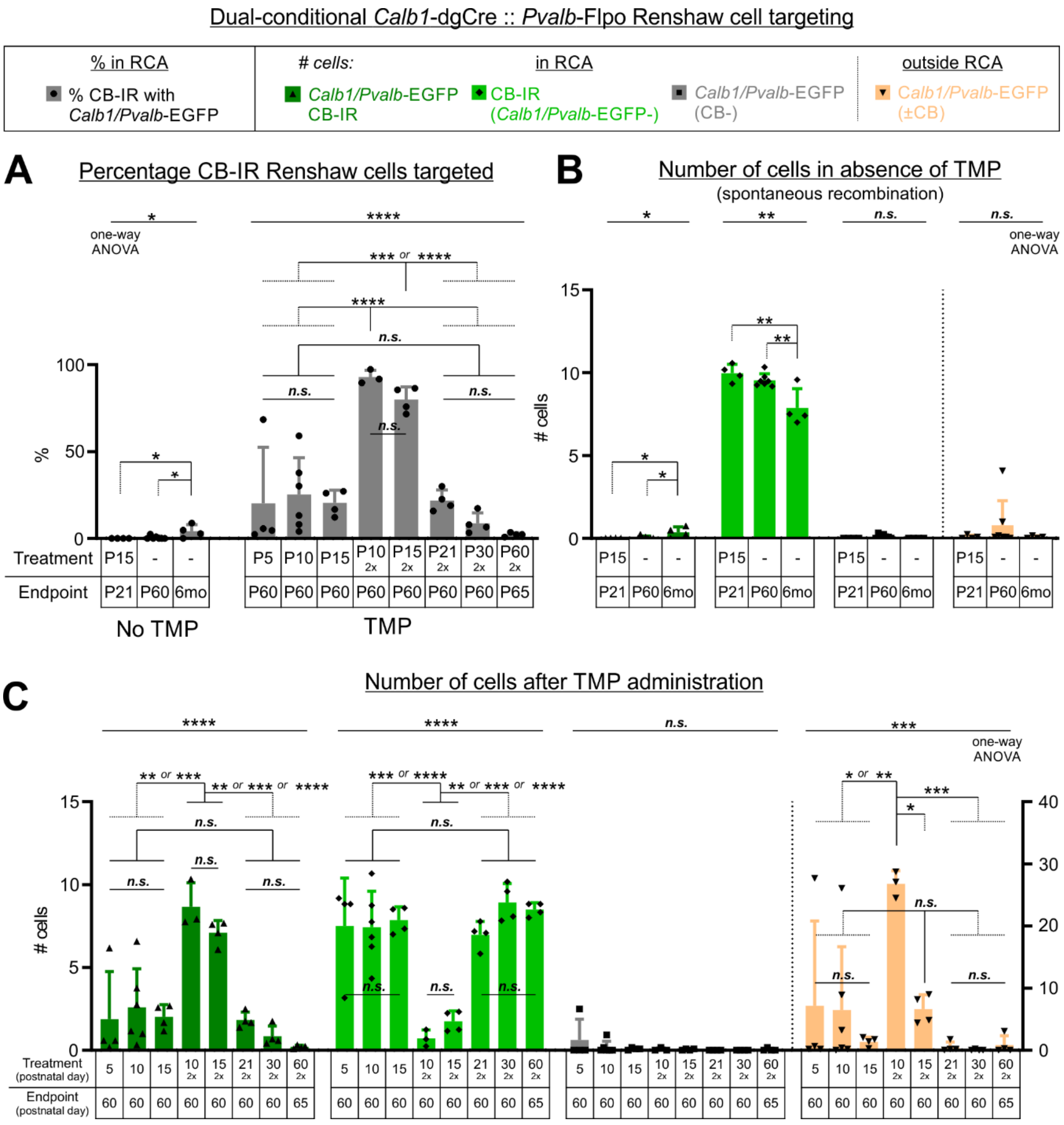

Supplemental Figure S4.

Percentages (A) and numbers (B, C) of CB-IR cells inside the RCA or outside the RCA that are genetically labeled by *Calb1/Pvalb*-EGFP without TMP (B) or following TMP administration (C) in *Calb1*-dgCre *::* *Pvalb*-Flpo *::* R26 *RCE:dual* animals. Each data point represents one animal. Statistical details (one-way ANOVAs followed by Bonferroni corrected multiple pair comparisons) are described in Supplemental Tables 7-9 as follows: spontaneous recombination in the RCA (Supplemental Table 7); spontaneous and TMP induced recombination outside the RCA (Supplemental Table 8); TMP-induced recombination in the RCA (Supplemental Table 9).

(**A**) **Percentage of CB-IR cells with *Calb1/Pvalb*-EGFP in the RCA**: In the absence of TMP, the percentage of CB-IR cells with *Calb1/Pvalb*-EGFP in the RCA at any age is negligible (<5%, left; Supplemental Table 7). One dose of TMP modestly increased the labeling of CB-IR cells with *Calb1/Pvalb*-EGFP (20-25%, right; Supplemental Table 9). Two doses of TMP at significantly increased targeting when administered at P10 or P15 (80-90%) but not when administered from P21 to P60 (Supplemental Table 9).

(**B**) **Number of cells targeted following spontaneous recombination in the absence of TMP**: In the absence of TMP, there is a slight increase in the number of *Calb1/Pvalb*-EGFP CB-IR cells in the RCA which is mirrored by a small decrease in the number of CB-IR cells lacking *Calb1/Pvalb*-EGFP (Supplemental Table 7). Dorsal to the RCA (any laminae) there were minimal numbers of *Calb1/Pvalb*-EGFP cells which did not change with animal age (Supplemental Table 8).

(**C**) **Number of cells targeted following TMP-induced recombination**:

Dark green: Successful targeting of Renshaw cells. Two injections of TMP at P10 or P15 significantly increases the numbers of CB-IR cells in the RCA expressing *Calb1*/*Pvalb*-EGFP (Supplemental Table 9).

Light green: Failed targeting of Renshaw cells. The number of CB-IR cells in the RCA lacking *Calb1*/*Pvalb*-EGFP mirror the results of the CB-IR cells expressing the genetic label (dark green bars) (Supplemental Table 9).

Gray: Off-target genetic labeling in the RCA. Negligible numbers of genetically labeled *Calb1*/*Pvalb*-EGFP cells in the RCA lacking CB-IR are found at any TMP injection time or dose (Supplemental Table 9).

Orange: Off-target genetic labeling dorsal to the RCA. Genetic *Calb1/Pvalb*-EGFP labeling of cells dorsal to the RCA is significantly higher after two injections at P10 compared to all other injection protocols; this includes two injections at P15, which also resulted in large number of Renshaw cells targeted (Supplemental Table 8).

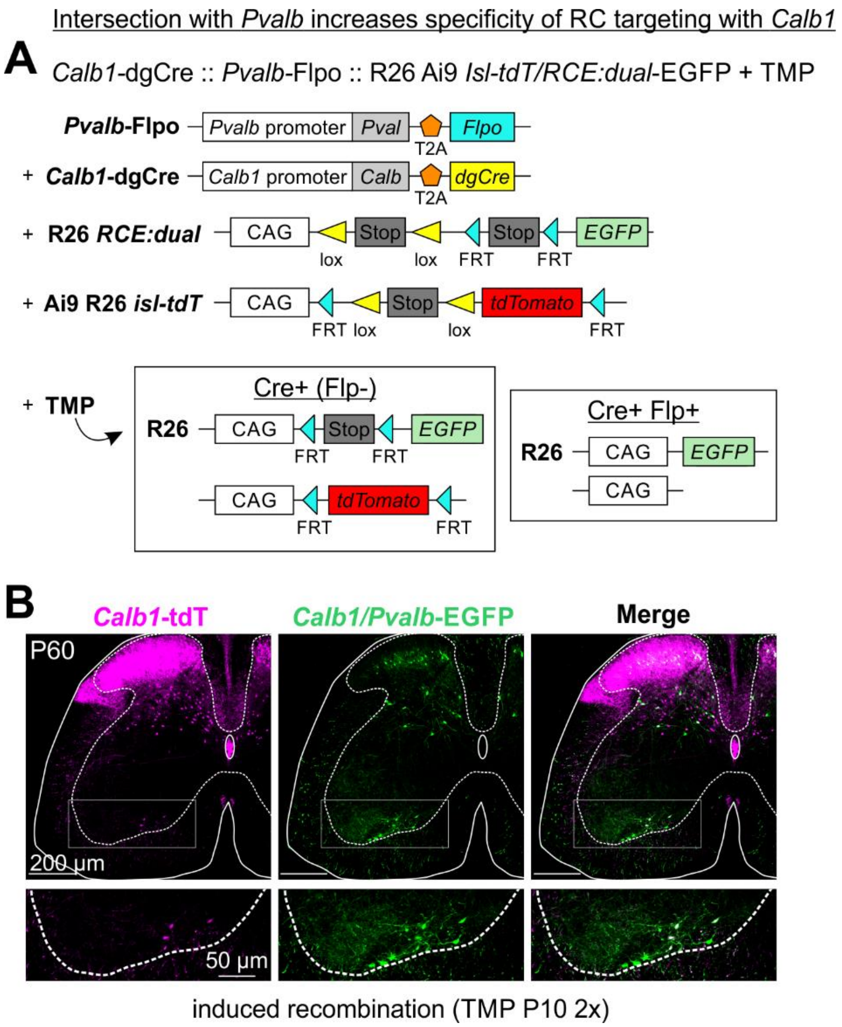

Supplemental Figure S5.

Analyses of *Calb1/Pvalb*-EGFP cells and *Calb1*-only tdT cells in *Calb1*-dgCre *::* *Pvalb*-Flpo *::* R26 *RCE:dual/lsl-tdT* animals.

(**A**) Genetic targeting design using two different reporter alleles in the R26 locus: Cre- and FlpO- dependent RCE:dual EGFP and Cre-dependent Ai9-tdTomato. Note that Flpo recombination also removes the tdTomato reporter cassette. Thus, cells expressing both recombinases will be tagged with EGFP, cells expressing only Cre will be tagged with tdTomato only, and cells expressing only Flp will not be tagged.

(**B**) Genetic reporter expression in a P60 animal after two TMP injections at P10. Many cells are tdT labeled (*Calb1*-tdT), which represent cells that underwent *Calb1*-dgCre recombination but not *Pvalb*-Flpo recombination. *Calb1/Pvalb*-EGFP cells underwent both *Calb1*-dgCre and *Pvalb*-Flpo recombination and thus expressed both *Calb1* and *Pvalb* during their lifetime. These cells include Renshaw cells in the ventral horn as well as superficial and deep layers of the dorsal horn. Some Renshaw cells co-express both tdTomato and EGFP (see higher magnification images). Coexpression of EGFP and tdTomato could be the result of comparatively late upregulation of *Pvalb*, Flpo recombination, and removal of the tdTomato reporter, with tdT protein transcribed prior to removal of the cassette lingering in the cell. Low levels of tdT are efficiently detected via immunostaining, which amplifies the signal.

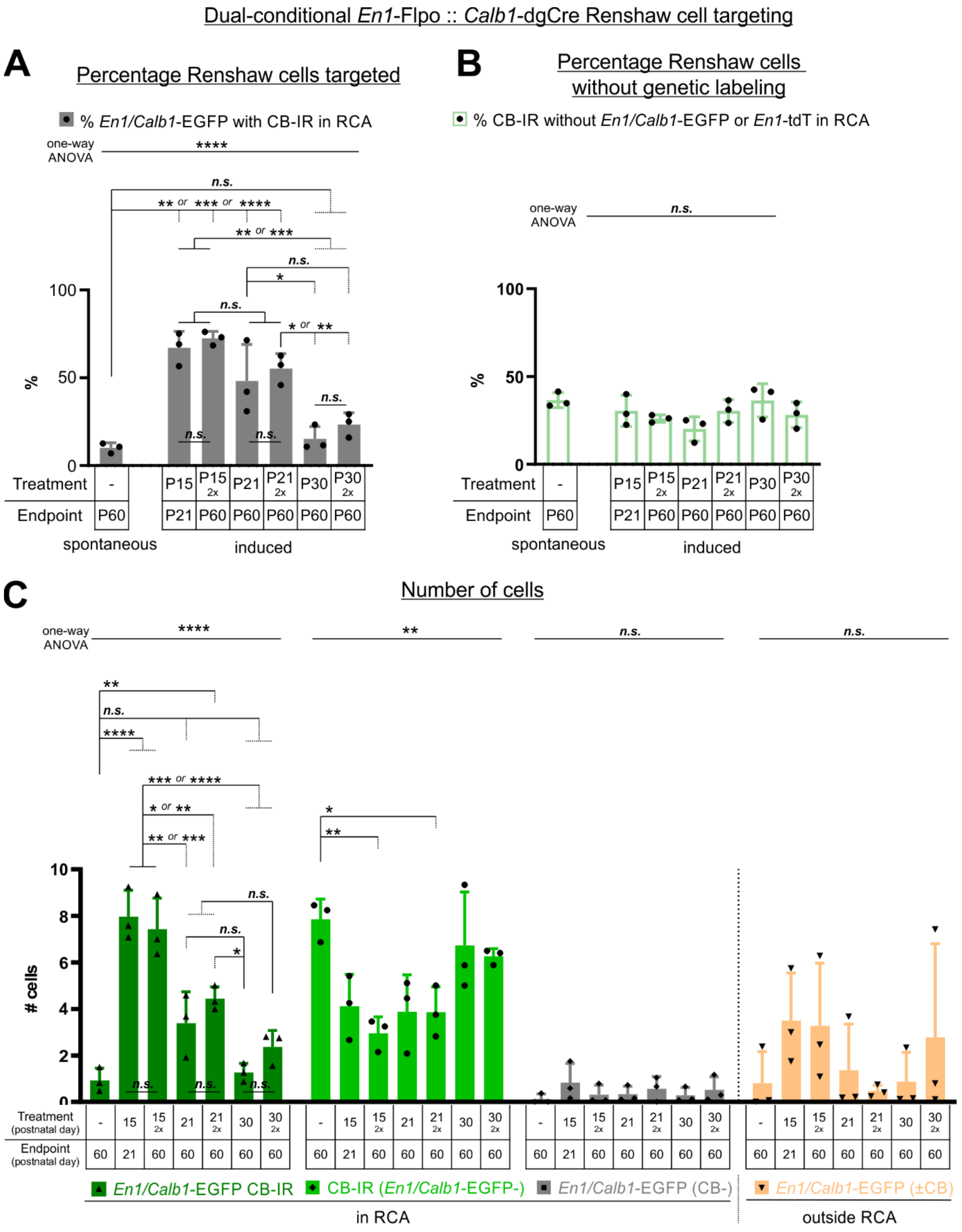

Supplemental Figure S6.

Percentages and numbers of Renshaw cells and non-Renshaw cells targeted in *Calb1*-dgCre *:: En1*-Flpo :: R26-FLTG animals.

With this genetic targeting design, cells expressing both recombinases will be tagged with EGFP (*En1/Calb1*-EGFP), cells expressing only Flpo will be tagged with tdTomato (*En1*-tdT), and cells expressing only Cre will not be tagged. Each data point represents one animal. Statistical details (one-way ANOVAs followed by Bonferroni corrected multiple pair comparisons) are described in Supplemental Tables 10-12.

(**A**) Percent of Renshaw cells targeted by *En1* and *Calb1* intersection: The percentage of *En1/Calb1*-EGFP cells with CB-IR is maximal when TMP is administered in one or two doses with P15 or P21 and is significantly lower when TMP is administered at P30. No significant differences were found between one or two doses (Supplemental Table 11).

(**B**) Percent of Renshaw cells that were not targeted by *En1* and *Calb1* intersection: The percentage of CB-IR cells that were not labeled with either *En1*-tdT or *En1/Calb1*-EGFP is constant across all TMP paradigms (Supplemental Table 11). Thus, these cells represent Renshaw cells which failed to undergo Flp recombination in the *En1-Flpo* animal.

(**C**) Numbers of different types of V1 cells labeled in the RCA and outside the RCA.

Dark green: Successful targeting of Renshaw cells. Greater numbers of CB-IR cells in the RCA with *En1/Calb1*-EGFP labeling are observed when TMP is administered at P15 and P21 than at P30 (Supplemental Table 10).

Light green: Failed targeting of Renshaw cells. The numbers of CB-IR cells in the RCA without *En1/Calb1*-EGFP labeling is lowest when TMP is administered at P15 and P21 (Supplemental Table 10).

Gray: Off-target genetic labeling in the RCA. There are very few *En1/Calb1*-EGFP cells lacking CB-IR in the RCA in all conditions, with no differences across TMP protocols (Supplemental Table 12).

Orange: Off-target genetic labeling dorsal to the RCA. There are few *En1/Calb1*-EGFP cells lacking CB-IR dorsal to the RCA; on average, approximately 1-4 cells were observed across all conditions, with no differences across TMP protocols (Supplemental Table 12).

<u>Supplemental Table 1</u> (accompany graph in Fig 1C)

Numbers *En1*-tdT (V1), *Mafb*-GFP, CB-IR cells at different ages

Age	animal #1	animal #2	animal #3	animal #4	Average ± S.D.
# ventral horns analyzed					
**P5**	2	4	4	4	**3.5 ± 1.0**
**P15**	12	12			**12**
**6 mo**	13	12	12		**12.3 ± 0.6**
in RCA area					
**P5**	13.0	12.3	11.5	11.3	**12.0 ± 0.8**
**P15**	8.8	7.8			**8.3 ± 0.7**
**6 mo**	6.4	6.9	6.5		**6.6 ± 0.3**
One-way ANOVA: F (2,6) = 64.68 ****$p < 0.0001$<br>Bonferroni's multiple comparison tests<br>**P5, P15** **$p = 0.0016$<br>**P5, 6 mo** ****$p < 0.0001$<br>**P15, 6 mo** $p = 0.0829$					
dorsal to the RCA area					
**P5**	0.5	1.3	0.5	0.8	**0.8 ± 0.4**
**P15**	0.4	1.3			**0.9 ± 0.7**
**6mo**	0.1	0.7	0.7		**0.5 ± 0.3**
One-way ANOVA: F (2,6) = 0.4791 $p = 0.6411$					

<u>Supplemental Table 2</u> (refer to graphs in Fig 2C)

Average number of cells in the RCA (per ventral horn) after injecting TMP in *Calb1*-dgCre/+ :: *Mafb*-GFP/+ :: Ai9 R26 *lsl-tdT*/+ animals

(each animal estimate is an average of 12 ventral horns)

**Animal #**	*Calb1*-tdT only	*Mafb*-GFP + only	CB-IR + only	*Mafb*-GFP + CB-IR (-) *Calb1*-tdT +	*Mafb*-GFP + CB-IR + *Calb1*-tdT (-)	*Mafb*-GFP + CB-IR + *Calb1*-tdT +
**Injected with 100 mg/kg TMP at P15 analyzed at P21**						
#1	3.3	0.1	0.3	0.0	1.8	9.5
#2	1.5	0.1	0.0	0.1	0.4	11.5
#3	0.0	0.0	0.3	0.0	1.3	10.3
#4	1.3	0.0	0.4	0.0	0.7	10.6
**Average ±S.D.**	**1.4 ±1.36**	**0.05 ±0.06**	**0.25 ±0.17**	**0.03 ±0.05**	**1.06 ±0.64**	**10.48 ±0.83**
**Injected with vehicle at P15 analyzed at P21**						
#1	7.8	0.9	0.2	0.2	8.8	0.0
#2	1.1	0.0	0.2	0.0	9.9	0.4
#3	1.0	0.0	0.0	0.0	9.3	0.1
#4	2.2	0.4	0.3	0.0	10.6	0.3
**Average ±S.D.**	**3.0 ±3.2**	**0.33 ±0.43**	**0.18 ±0.13**	**0.05 ±0.10**	**9.7 ±0.75**	**0.19 ±0.18**

Supplemental Table 3 (refer to graphs in Fig 2D and S2A)

RCs (CB-IR cells in RCA) expressing *Calb1*-tdT after different TMP doses at P15 in *Calb1* dgCre/+ :: Ai9 R26 *lsl-tdT*/+ animals

(12 ventral horns analyzed per animal)

	**TMP 50 mg/kg**		**TMP 100 mg/kg**		**TMP 125 mg/kg**	
Total number RCs sampled and % of RCs expressing tdT in Calb1-tdT animals						
**Animal #**	**# RCs**	**%**	**# RCs**	**%**	**# RCs**	**%**
#1	126	76.0	135	85.1	135	93.7
#2	146	79.1	123	86.8	151	92.2
#3	143	83.4	122	81.8	144	93.0
#4	129	86.4	105	93.2	109	89.7
#5			141	83.0		
#6			143	96.8		
#7			143	86.3		
#8			140	90.2		
**Average ± S.D.**		**81.2 ± 4.6**		**87.9 ± 5.2**		**92.2 ± 1.7**

Statistics; % of RCs expressing *Calb1*-tdT
One Way ANOVA F (2, 13) = 6.146, ***p = 0.0132****
Post-hoc Bonferroni's multiples comparisons
50 vs 100 t(13) = 2.443 p = 0.0888
50 vs 125 t(13) = 3.464 ***p = 0.0126****
100 vs 125 t(13) = 1.556 p = 0.4308

***Column A**: Average number of RCs sampled per ventral horn*
***Column B**: Average number of RCs lacking Calb1-tdT genetic labeling*
***Column C**: Average number of Calb1-tdT cells in RCA lacking CB-IR*

	**TMP 50 mg/kg**			**TMP 100 mg/kg**			**TMP 125 mg/kg**		
**Animal #**	**A**	**B**	**C**	**A**	**B**	**C**	**A**	**B**	**C**
#1	10.5	2.4	0.9	11.3	1.6	1.1	11.3	0.8	0.6
#2	12.2	2.6	1.3	10.3	1.3	1.3	11.6	0.9	3.8
#3	11.9	2.3	2.6	10.2	1.9	2.9	12.0	0.9	0
#4	10.8	1.6	0.8	9.6	0.6	0.4	9.1	0.8	0
#5				11.8	2.1	3.3			
#6				11.9	0.4	1.5			
#7				11.9	1.6	0			
#8				11.7	1.1	1.3			
**Average ± S.D.**	**11.3 ±0.8**	**2.2 ±0.4**	**1.4 ±0.8**	**11.0 ± 0.7**	**1.1 ±0.6**	**1.4 ±1.1**	**11.0 ±1.3**	**0.86 ±0.04**	**1.1 ±1.8**

Statistics Column A: Average number of RCs sampled
One Way ANOVA F (2, 13) = 0.1373, p = 0.8729
Statistics Column B: Average number of RCs lacking *Calb1*-tdT
One Way ANOVA F (2, 13) = 8.314, ***p = 0.0047*****
Post-hoc Bonferroni's multiples comparisons
50 vs 100 t(13) = 3.025 ***p = 0.0293****
50 vs 125 t(13) = 3.979 ***p = 0.0047*****
100 vs 125 t(13) = 1.570 p = 0.4214
Statistics Column C: Average number of *Calb1*-tdT cells in RCA lacking CB-IR
One Way ANOVA F (2, 13) = 0.1185, p = 0.8892

<u>Supplemental Table 4</u> (refer to graphs in Fig 2E and S2B)

***Calb1*-tdT labeling of CB-IR cells in the RCA after TMP injections at different time points in *Calb1 dgCre*/+ :: Ai9 R26 *lsl-tdT*/+ animals**

(data are average numbers of cells per RCA, 11-13 RCAs analyzed per animal)

	Animal #	#1	#2	#3	#4	#5	#6	#7	#8	**Average ±S.D.**
All *Calb1*-tdT *(cells per RCA)*	P5 → P21	5.7	13.5	15.7	13.4	15.2				12.7 ±4.1
	P10 → P21	10.8	13.6	12.9	14.4					12.9 ±1.5
	P15 → P21	10.8	10.3	11.2	9.3	13.0	13.0	10.3	11.8	11.2 ±1.3
	P15 → P60	11.4	8.3	7.8	11.1					9.7 ±1.8
	P60 → P65	2.6	4.9	3.4	3.4					3.6 ±0.97
	One-way ANOVA: $F(4,20) = 12.45$ ***p < 0.0001**** Bonferroni's multiple comparison tests; **P60 → P65** vs P5 → P21 ***p < 0.0001******; **P60 → P65** vs P10 → P21 ***p < 0.0001******; **P60 → P65** vs P15 → P21 ***p = 0.0002*****; **P60 → P65** vs P15 → P60 ***p = 0.0093****; All other pair comparisons not significant									
All CB-IR+ *(cells per RCA)*	P5 → P21	8.3	11.5	10.4	12.1	10.8				10.6 ±1.5
	P10 → P21	10.3	13.7	10.2	11.3					11.4 ±1.6
	P15 → P21	11.3	10.3	10.2	9.6	11.8	11.9	11.9	11.7	11.1 ±0.9
	P15 → P60	11.1	9.3	8.7	11.0					10.0 ±1.2
	P60 → P65	7.5	8.9	8.5	8.9					8.5 ±0.66
	One-way ANOVA: $F(4,20) = 4.071$ ***p = 0.0142**** Bonferroni's multiple comparison tests; **P60 → P65** vs P10 → P21 ***p = 0.0255****; **P60 → P65** vs P15 → P21 ***p = 0.0191****; All other pair comparisons not significant									
CB-IR+ and *Calb1*-tdT *(cells per RCA)*	P5 → P21	5.5	11.4	10.4	11.9	10.8				10.0 ±2.6
	P10 → P21	10.3	13.5	10.0	11.2					11.3 ±1.6
	P15 → P21	9.7	9.0	8.3	8.9	9.7	11.5	10.3	10.6	9.7 ±1.0
	P15 → P60	9.8	8.3	7.7	9.5					8.8 ±1.0
	P60 → P65	1.8	4.5	3.3	3.3					3.2 ±1.1
	One-way ANOVA: $F(4,20) = 16.79$ ***p < 0.0001****** Bonferroni's multiple comparison tests; **P60 → P65** vs P5 → P21, P10 → P21, P15 → P21, or P15 → P60; all ***p < 0.0001******; All other pair comparisons not significant									
% CB-IR+ with *Calb1*-tdT	P5 → P21	67.5	99.4	100.0	98.8	100.0				93.1 ±0.14
	P10 → P21	100.0	98.6	98.3	99.5					99.1 ±0.01
	P15 → P21	85.1	86.8	81.8	93.2	83.0	96.8	86.3	90.2	87.9 ±0.05
	P15 → P60	89.6	90.7	88.0	86.3					88.6 ±0.02
	P60 → P65	24.4	50.6	40.8	37.1					38.2 ±0.11
	One-way ANOVA: $F(4,20) = 35.94$ ***p < 0.0001****** Bonferroni's multiple comparison tests; **P60 → P65** vs P5 → P21, P10 → P21, P15 → P21, or P15 → P60; all ***p < 0.0001******; All other pair comparisons not significant									
Calb1-tdT that are CB-IR(-) *(cells per RCA)*	P5 → P21	0.17	2.08	5.25	1.46	4.42				2.7 ±2.1
	P10 → P21	0.50	0.08	2.92	3.25					1.7 ±1.6
	P15 → P21	1.08	1.25	2.92	0.36	3.33	1.50	0	1.25	1.5 ±1.2
	P15 → P60	1.58	0	0.2	1.6					0.83 ±0.87
	P60 → P65	0.83	0.42	0.17	0.08					0.38 ±0.34
	One-way ANOVA: $F(4,20) = 1.850$ $p = 0.1589$									

<u>Supplemental Table 5</u> **(refer to graphs in Fig 2E and S2C)**

Spontaneous recombination in RCs and non-RCs in the RCA at different ages in *Calb1 dgCre/+* :: Ai9 R26 *lsl-tdT/+* animals

(data are average numbers of cells per RCA: 6 RCAs analyzed per animal at P5, 8-12 RCAs analyzed per animal for other timepoints)

	Animal #	#1	#2	#3	#4	#5	#6	#7	#8	#9	#10	#11	#12	**Average ±S.D.**
CB-IR+ and *Calb1*-tdT<br><br>spont. recomb. in RCs	P5	0	0	0	0									0
	P21	0.1	0.4	0.2	0.0	0.3	0.3	1.6	0.6	0	0.4	0.1	0.3	0.35 ±0.44
	P60	1.5	1.4	0.9	1.7	1.4	1.4	1.2	1.1					1.3 ±0.25
	6 mo.	3.8	5.7	4.1	6.3									5.0 ±1.2
	One-way ANOVA: F (3,24) = 81.79 ***p < 0.0001*******<br>Bonferroni's multiple comparison tests<br>**6 months** vs P5, P21, P60, ***p < 0.0001*******<br>**P60** vs P5, ***p = 0.0031*****; vs P21, ***p = 0.0036*****<br>P5 vs P21 not significant (p > 0.9999)													
CB-IR+ only<br><br>No spont. recomb. in RCs	P5	12.5	13.7	16.3	12.7									13.8 ±1.8
	P21	11.5	10.9	11.3	11.3	8.5	11.8	11.5	9.0	9.0	10.1	9.3	10.8	10.4 ±1.2
	P60	7.1	7.6	6.1	8.7	4.8	7.6	7.5	7.2					7.1 ±1.2
	6 mo.	6.2	3.5	2.7	3.8									4.0 ±1.5
	One-way ANOVA: F (3,24) = 48.42 ***p < 0.0001*******<br>Bonferroni's multiple comparison tests<br>**6 months** vs P5, P21, ***p < 0.0001****;*** vs P60, ***p = 0.0052*****<br>**P60** vs P5, P21, ***p < 0.0001*******<br>**P21** vs P5, ***p = 0.0008******													
Calb1-tdT only<br><br>spont. recomb. in non-RCs	P5	0	0.8	4.2	0.7									1.4 ±1.9
	P21	0	0.1	0.2	2.1	2.2	0.4	3.3	0.6	7.8	1.1	1.0	2.2	1.7 ±2.2
	P60	2.6	0	1.8	0.2	0.3	3.0	3.6	4.7					2.0 ±1.7
	6 mo.	0.3	1.1	0.7	0.2									0.56 ±0.4
	One-way ANOVA: F (3,24) = 0.5698 p = 0.6403													
All CB-IR+	P5	12.5	13.7	16.3	12.7									13.8 ±1.8
	P21	11.6	11.3	11.4	11.3	8.8	12.1	13.1	9.6	9.0	10.5	9.4	11.1	10.8 ±1.3
	P60	8.5	9.0	7.3	9.8	6.3	9.0	8.4	8.8					8.4 ±1.1
	6 mo.	10.0	9.2	6.8	10.2									9.0 ±1.6
	One-way ANOVA: F (3,24) = 15.58 ***p < 0.0001*******<br>Bonferroni's multiple comparison tests<br>**6 months** vs P5, ***p = 0.0003******<br>**P60** vs P5, ***p < 0.0001*******; vs P21, ***p = 0.0051*****<br>**P21** vs P5, ***p = 0.0085*****<br>6 months vs P21 (p = 0.2226) or P60 (p > 0.9999), not significant													
% CB-IR with *Calb1*-tdT<br><br>% spont. recomb. in RCs	P5	0	0	0	0									0
	P21	0.6	4.4	1.5	0	3.7	3.0	11.7	6.1	0	4.6	0.9	2.0	3.2 ±3.3
	P60	16.4	15.5	16.1	11.5	23.9	16.6	10.9	18.8					16.2 ±4.1
	6 mo.	40.0	63.1	55.1	64.3									55.6 ±11.2
	One-way ANOVA: F (3,24) = 118.98 ***p < 0.0001*******<br>Bonferroni's multiple comparison tests<br>**6 months** vs P5, P21, P60, ***p < 0.0001*******<br>**P60** vs P5, ***p < 0.0001*******; vs P21 ***p = 0.0001*******<br>P5 vs P21 not significant (p > 0.9999)													

<u>Supplemental Table 6</u> (refer to graphs in Fig 2F,G and S3)

Numbers and percentages of *Calb1*-tdT cells expressing CR-IR or PV-IR after TMP administration in *Calb1 dgCre*/+ :: Ai9 R26 *lsl-tdT*/+ animals

(data are average numbers of cells per RCA, 7-12 RCAs analyzed per animal)

	Animal #	1	2	3	4	5	6	7	8	9	10	11	**Average ±S.D.**
Calb1-tdT without CR	P5 → P21	1.7	5.3	5.0	4.7	6.3							4.58 ±1.74
	P10 → P21	5.1	6.0	5.5									5.54 ±0.44
	P15 → P21	3.4	3.4	3.3	3.0	3.7	5.5	5.5	4.4	4.6	3.9	2.8	3.96 ±0.95
	P15 → P60	4.3	2.0	2.7	2.0								2.75 ±1.10
	One-way ANOVA: F (3,19) = 3.769 ***p = 0.0281**** Bonferroni's multiple comparison tests **P10 → P21** vs **P15 → P60** ***p = 0.0292****												
Calb1-tdT with CR	P5 → P21	2.1	7.5	9.2	8.8	5.5							6.63 ±2.91
	P10 → P21	4.8	7.8	8.4									6.97 ±1.95
	P15 → P21	5.1	5.3	9.0	7.0	5.0	4.1	5.4	4.8	5.6	7.9	8.0	6.11 ±1.6
	P15 → P60	3.9	5.9	6.0	5.7								5.38 ±0.98
	One-way ANOVA: F (3,19) = 0.4976 p = 0.6883												
% of cells *Calb1*-tdT with CR	P5 → P21	46.7	58.8	65.8	66.5	50.4							57.62 ±8.93
	P10 → P21	50.8	57.8	58.9									55.79 ±4.4
	P15 → P21	56.9	63.4	70.8	71.8	56.5	46.8	46.5	52.3	56.6	67.2	74.2	60.26 ±9.85
	P15 → P60	46.3	74.9	68.7	73.2								65.76 ±13.23
	One-way ANOVA: F (3,19) = 0.7344 p = 0.5443												
Calb1-tdT without PV	P5 → P21	5.7	9.3	3.8									6.24 ±2.82
	P10 → P21	4.3	3.8	6.7									4.94 ±1.51
	P15 → P21	4.3	5.2	3.0	5.9	4.4	7.1	2.7	1.5				4.25 ±1.85
	One-way ANOVA: F (2,11) = 1.074 p = 0.3750												
Calb1-tdT with PV	P5 → P21	7.0	6.6	9.8									7.8 ±1.74
	P10 → P21	6.3	9.1	7.8									7.73 ±1.4
	P15 → P21	3.3	7.6	6.6	5.1	4.9	10.7	8.4	6.9				6.69 ±2.3
	One-way ANOVA: F (2,11) = 0.4635 p = 0.6408												
% of cells *Calb1*-tdT with PV	P5 → P21	55.0	41.0	73.3									56.45 ±16.18
	P10 → P21	58.8	72.5	55.0									62.11 ±9.22
	P15 → P21	49.3	58.6	70.8	44.1	50.1	59.4	74.6	80.8				60.98 ±13.22
	One-way ANOVA: F (2,11) = 0.1662 p = 0.8490												

Supplemental Table 7 (refer to graphs in Fig 3C and S4A,B)

Cells in the RCA targeted by *Calb1/Pvalb* intersection without TMP (spontaneous) in *Calb1* dgCre/+ :: *Pvalb*-Flpo/+ :: R26 *RCE:dual*/+ animals

(data are average numbers of cells per RCA, 8-12 RCAs analyzed per animal)

	Animal # / Analysis Date	#1	#2	#3	#4	#5	#6	#7	**Average ±S.D.**
EGFP+ CB-IR RCs	P21	0	0	0	0				0
	P60	0	0.25	0	0.08	0	0	0	0.05 ±0.09
	6 months	0	0.50	0.75	0.25				0.38 ±0.32
	One-way ANOVA: F (2,12) = 5.819 ***p = 0.0171****<br>Bonferroni's multiple comparison tests (all other comparisons not significant)<br>**6 months** vs P21 ***p = 0.0310****<br>vs P60 ***p = 0.0335****								
EGFP+ CB-IR neg non-RCs	P21	0	0	0	0				0 ±0
	P60	0.08	0.33	0	0	0	0.25	0	0.1 ±0.14
	6 months	0	0	0	0				0 ±0
	One-way ANOVA: F (2,12) = 10.64 p = 0.2199								
EGFP neg CB-IR RCs	P21	9.8	10.6	10.2	9.3				9.98 ±0.53
	P60	9.3	9.5	9.8	9.3	10.3	9.5	9.2	9.55 ±0.4
	6 months	9.6	7.5	7.4	7.0				7.88 ±1.16
	One-way ANOVA: F (2,12) = 5.819 ***p = 0.0022*****<br>Bonferroni's multiple comparison tests (all other comparisons not significant)<br>**6 months** vs P21 ***p = 0.0032*****<br>vs P60 ***p = 0.0071*****								
% CB-IR RCs EGFP+	P21	0	0	0	0				0
	P60	0	2.45	0	0.93	0	0	0	0.48 ±0.94
	6 months	0	4.84	8.88	2.84				4.14 ±3.73
	One-way ANOVA: F (2,12) = 5.574 ***p = 0.0194****<br>Bonferroni's multiple comparison tests (all other comparisons not significant)<br>**6 months** vs P21 ***p = 0.0359****<br>vs P60 ***p = 0.0366****								

<u>Supplemental Table 8</u> (refer to graphs in Fig 3C and S4)

TMP-induced and spontaneous *Calb1/Pvalb*-EGFP labeling of cells dorsal to the RCA in *Calb1*-dgCre/+ :: *Pvalb*-Flpo/+ :: R26 *RCE:dual*/+ animals

(data are average numbers of cells per hemicord, 8-12 hemicords analyzed per animal; P60 2x was analyzed at P65, all other animals were analyzed at P60)

EGFP labeled cells after different TMP injections

	Animal # / TMP injection	#1	#2	#3	#4	#5	#6	Average ±S.D.
EGFP+ Non-RCs	P5	27.7	0.7	0.2	0.2			7.17 ±13.67
	P10	8.9	26.1	0.2	0	0.4	3.3	6.47 ±10.19
	P15	0.3	2.1	1.8	1.4			1.4 ±0.76
	P10 2x	24.5	27.1	28.8				26.78 ±2.14
	P15 2x	9.0	4.8	8.2	4.4			6.6 ±2.32
	P20 2x	1.8	0.1	0.2	0.2			0.54 ±0.81
	P30 2x	0.3	0	0	0			0.06 ±0.13
	P60 2x	0	0	3.1	0.3			0.85 ±1.49

One-way ANOVA: $F_{(7,25)} = 5.603$ ***p = 0.0006******

Bonferroni's multiple comparison tests (all other comparisons not significant)

P10 2x vs P5 → P60, ***p = 0.0207****
vs P10 → P60, ***p = 0.0064*****
vs P15 → P60, ***p = 0.0011*****
vs P15 2x → P60, ***p = 0.0156****
vs P21 2x → P60, ***p = 0.0007******
vs P30 2x → P60, ***p = 0.0006******
vs P60 2x → P65, ***p = 0.0008******

EGFP labeled cells without TMP (spontaneous)

	Animal # / Analysis Date	#1	#2	#3	#4	#5	#6	#7	Average ±S.D.
EGFP+ Non-RCs	P21	0.08	0.25	0	0				0.08 ±0.12
	P60	0.08	1.00	0	0.17	0.08	4.08	0.08	0.79 ±1.49
	3 months	0.17	0.1	0	0				0.06 ±0.08

One-way ANOVA: $F_{(2,12)} = 0.8441$ $p = 0.4539$

Supplemental Table 9 (refer to graphs in Fig 3C and S4A,C)

Cells in the RCA targeted by *Calb1/Pvalb* intersection after TMP injections in *Calb1*-dgCre/+ :: *Pvalb*-Flpo/+ :: R26 *RCE:dual*/+ animals

(data are average numbers of cells per RCA, 8-12 RCAs analyzed per animal; P60 2x was analyzed at P65, all other animals were analyzed at P60)

	Animal # / TMP injection	#1	#2	#3	#4	#5	#6	**Average ±S.D.**
EGFP+ CB-IR RCs	P5	6.2	0.6	0.2	0.5			1.85 ±2.88
	P10	2.8	6.6	1.0	0.3	1.2	3.8	2.6 ±2.32
	P15	1.2	2.6	2.7	1.7			2.02 ±0.73
	P10 2x	7.8	7.9	10.3				8.67 ±1.45
	P15 2x	7.6	7.1	7.7	6.1			7.1 ±0.73
	P20 2x	2.5	1.4	1.7	1.8			1.83 ±0.48
	P30 2x	0.7	0.6	0.4	1.8			0.85 ±0.61
	P60 2x	0.1	0.3	0.3	0.2			0.21 ±0.11
	One-way ANOVA: F (7,25) = 13.43 ***p < 0.0001******* Bonferroni's multiple comparison tests (all other comparisons are not significant)<br>**P10 2x** vs P5, P15, P21 2x ***p = 0.0002******; vs P10 ***p = 0.0003***;*** vs P30 2x, P60 2x ***p < 0.0001*******<br>**P15 2x** vs P5, ***p = 0.0021*****; vs P10 ***p = 0.0040******; vs P15 ***p = 0.0029*****; vs P21 2x ***p = 0.0018*****;<br>vs P30 2x ***p = 0.0002*****; vs P60/61 ***p < 0.0001*******							
EGFP+ CB-IR neg non-RCs	P5	2.5	0	0	0			0.63 ±1.25
	P10	0.2	0.9	0	0	0	0	0.18 ±0.37
	P15	0	0.2	0.1	0.1			0.08 ±0.07
	P10 2x	0.2	0	0				0.06 ±0.1
	P15 2x	0	0.1	0.1	0			0.04 ±0.05
	P20 2x	0	0	0	0			0
	P30 2x	0	0	0	0			0
	P60 2x	0	0	0.2	0			0.04 ±0.08
	One-way ANOVA: F (7,25) = 0.8023 p = 0.5931							
EGFP neg CB-IR RCs	P5	3.2	8.8	8.8	9.2			7.5 ±2.89
	P10	6.3	4.3	10.7	8.8	7.8	6.8	7.43 ±2.18
	P15	8.5	7.3	7.0	8.6			7.85 ±0.81
	P10 2x	0.3	0.7	1.3				0.72 ±0.5
	P15 2x	1.2	2.3	1.3	2.3			1.75 ±0.63
	P20 2x	5.8	7.8	7.2	7.1			6.96 ±0.81
	P30 2x	9.6	7.8	10.2	8.1			8.92 ±1.14
	P60 2x	8.0	8.8	8.8	8.3			8.5 ±0.41
	One-way ANOVA: F (7,25) = 15.00 ***p < 0.0001******* Bonferroni's multiple comparison tests (all other comparisons are not significant)<br>**P10 2x** vs P5, ***p = 0.0001******; vs P21 2x ***p = 0.0002******; vs P10, P15, P30 2x, P60 2x ***p < 0.0001*******<br>**P15 2x** vs P5, ***p = 0.0005*****; **vs** P10, P15, ***p = 0.0002******; vs P21 2x ***p = 0.0017*****;<br>vs P30 2x, P60 2x ***p < 0.0001*******							
% CB-IR RCs EGFP+	P5	68.5	4.6	2.3	5.7			20.26 ±32.17
	P10	30.2	59.2	8.1	3.9	13.2	37.7	25.37 ±21.09
	P15	12.2	26.1	27.2	16.7			20.53 ±7.26
	P10 2x	97.3	91.8	89.6				92.92 ±3.98
	P15 2x	86.5	75.8	85.5	71.7			79.88 ±7.31
	P20 2x	30.0	15.8	19.0	22.6			21.86 ±6.11
	P30 2x	7.9	6.6	3.3	17.3			8.76 ±6
	P60 2x	0.8	3.5	2.2	2.3			2.18 ±1.12
	One-way ANOVA: F (7,25) = 16.80 ***p < 0.0001******* Bonferroni's multiple comparison tests (all other comparisons are not significant)<br>**P10 2x** vs P15 2x p > 0.9999; vs. all others ***p < 0.0001*******<br>**P15 2x** vs P5, P10, P15 ***p = 0.0003******; vs P21 2x ***p = 0.0004******; vs P30 2x, P60 2x ***p < 0.0001*******							

<u>Supplemental Table 10</u> (refer to graphs in Fig 5C and S6)

Numbers of V1 cells in the RCA targeted by *Calb1/En1* intersection after TMP injections in *Calb1* dgCre/+ :: *En1*-Flpo/+ :: R26 FLTG animals

(data are average numbers of cells per RCA, 8-22 RCAs analyzed per animal; all animals were analyzed at P60 unless otherwise specified)

	TMP injection → analysis / Animal #	P60	P15 → P21	P15 2x	P21	P21 2x	P30	P30 2x
All CB-IR RCs	#1	9.3	10.3	12.4	6.4	8.8	6.7	9.3
	#2	7.3	13.5	10.3	8.8	7.1	10.6	9.2
	#3	9.8	12.5	8.5	6.7	9.0	6.8	7.4
	**Average ± S.D.**	**8.8 ±1.3**	**12.1 ±1.7**	**10.4 ±1.9**	**7.3 ±1.3**	**8.3 ±1**	**8.0 ±2.2**	**8.6 ±1**
	One-way ANOVA: F (6,14) = 3.303 ***p = 0.0307****<br>Bonferroni's multiple comparison tests (all other comparisons are not significant)<br>**P15 → P21** vs P21 ***p = 0.0424****							
EGFP+ CB-IR RCs	#1	0.8	7.6	8.9	1.9	5.0	1.7	2.8
	#2	0.5	9.3	7.0	3.7	4.3	1.3	2.8
	#3	1.5	7.1	6.4	4.6	4.0	0.9	1.6
	**Average ± S.D.**	**0.9 ±0.5**	**8.0 ±1.1**	**7.4 ±1.3**	**3.4 ±1.4**	**4.4 ±0.5**	**1.3 ±0.4**	**2.4 ±0.7**
	One-way ANOVA: F (6,14) = 27.32 ***p < 0.0001*******<br>Bonferroni's multiple comparison tests (all other comparisons are not significant)<br>**P60** vs P15 → P21, P15 2x , ***p < 0.0001*******; vs P21 2x ***p = 0.0086*****<br>**P15 → P21** vs P21 ***p = 0.0007******; P21 2x ***p = 0.0081*****; vs P30, P30 2x ***p < 0.0001*******<br>**P15 2x** vs P21 ***p = 0.0024*****; vs P21 2x ***p = 0.0325****; vs P30 ***p < 0.0001*******; vs P30 2x ***p = 0.0002******<br>**P21 2x** vs P30 ***p = 0.0201*****							
CB-IR, EGFP- RCs	#1	8.5	2.7	3.5	4.5	3.8	5.0	6.5
	#2	6.9	4.3	3.3	5.1	2.8	9.3	6.4
	#3	8.3	5.4	2.1	2.1	5.0	5.9	5.9
	**Average ± S.D.**	**7.9 ±0.9**	**4.1 ±1.4**	**3.0 ±0.7**	**3.9 ±1.6**	**3.9 ±1.1**	**6.7 ±2.3**	**6.3 ±0.3**
	One-way ANOVA: F (6,14) = 5.788 ***p = 0.0033*****<br>Bonferroni's multiple comparison tests (all other comparisons are not significant)<br>**P60** vs. P15 2x ***p = 0.0097*****; vs P21 2x ***p = 0.0495****							
tdT+ CB-IR RC	#1	4.8	0.3	0.1	2.7	0.7	2.2	4.7
	#2	4.2	0.3	0.3	2.9	0.5	6.7	3.8
	#3	4.4	0.5	0.0	1.0	2.9	3.1	3.2
	**Average ± S.D.**	**4.5 ±0.3**	**0.3 ±0.1**	**0.1 ±0.2**	**2.2 ±1**	**1.4 ±1.3**	**4 ±2.4**	**3.9 ±0.7**
	One-way ANOVA: F (6,14) = 7.405 ***p = 0.0010*****<br>Bonferroni's multiple comparison tests (all other comparisons are not significant)<br>**P60** vs P15 → P21 ***p = 0.0125****; P15 2x, ***p = 0.0084*****<br>**P15 → P21** vs P30 ***p = 0.0337****; P30 2x ***p = 0.0408****<br>**P15 2x** vs P30 ***p = 0.0224****; P30 2x ***p = 0.0270****							
CB-IR RCs No TdT No EGFP	#1	3.6	2.4	3.4	1.7	3.1	2.8	1.8
	#2	2.7	4.0	2.9	2.2	2.3	2.7	2.6
	#3	3.9	4.9	2.1	1.1	2.1	2.8	2.7
	**Average ± S.D.**	**3.4 ±0.6**	**3.8 ±1.3**	**2.8 ±0.6**	**1.7 ±0.6**	**2.5 ±0.5**	**2.8 ±0.1**	**2.4 ±0.5**
	One-way ANOVA: F (6,14) = 3.079 ***p = 0.0389****<br>Bonferroni's multiple comparison tests (all other comparisons are not significant)<br>**P15 → P21** vs P21 ***p = 0.0417****							

<u>Supplemental Table 11</u> (refer to graphs in Fig 5C and S6)

Percentage of V1 cells in the RCA targeted by *Calb1/En1* intersection after TMP injections in *Calb1*-dgCre/+ :: *En1*-Flpo/+ :: R26 FLTG animals

(data are average numbers of cells per RCA, 8-22 RCAs analyzed per animal;
all animals were analyzed at P60 unless otherwise specified)

	TMP injection → analysis<br>Animal #	P60	P15	P15 2x	P21	P21 2x	P30	P30 2x
% CB-IR RCs EGFP+	#1	10.8	75.2	73.1	31.0	57.4	23.4	29.2
	#2	6.9	69.2	68.4	42.1	62.5	11.5	25.0
	#3	12.6	56.7	76.2	71.5	45.8	10.7	16.0
	**Average ± S.D.**	**10.1 ±2.9**	**67.0 ±9.5**	**72.6 ±3.9**	**48.2 ±20.9**	**55.2 ±8.6**	**15.2 ±7.1**	**23.4 ±6.8**
	One-way ANOVA: $F(6,14) = 18.74$ ***$p < 0.0001$***** Bonferroni's multiple comparison tests<br>**P60** vs P15 → P21 , ***$p = 0.0002$****; vs P15 2x, ***$p < 0.0001$*****; vs P21 ***$p = 0.0087$***<br>vs P21 2x , ***$p = 0.0018$***<br>**P15 → P21** vs P30 ***$p = 0.0004$***; P30 2x ***$p = 0.0025$***<br>**P15 2x** vs P30 ***$p = 0.0001$****; P30 2x ***$p = 0.0008$****<br>**P21** vs P30 ***$p = 0.0286$**<br>**P21 2x** vs P30 ***$p = 0.0055$***; P30 2x ***$p = 0.0374$**							
% CB-IR RCs tdT+	#1	54.4	2.3	0.7	45.8	6.1	34.1	50.4
	#2	59.6	2.1	3.5	32.8	6.3	63.0	45.8
	#3	45.9	3.4	0.0	16.2	30.7	48.1	49.0
	**Average ± S.D.**	**53.3 ±6.9**	**2.6 ±0.7**	**1.4 ±1.8**	**31.6 ±14.9**	**14.4 ±14.1**	**48.4 ±14.5**	**48.4 ±2.4**
	One-way ANOVA: $F(6,14) = 15.38$ ***$p < 0.0001$***** Bonferroni's multiple comparison tests<br>**P60** vs P15, ***$p = 0.0004$****; vs P15 2x, ***$p = 0.0003$****; vs P21 2x, ***$p = 0.0058$***<br>**P15 → P21** vs P30, P30 2x, ***$p = 0.0012$****<br>**P15 2x** vs P21, ***$p = 0.0468$**; vs P30, P30 2x, ***$p = 0.0010$***<br>**P21 2x** vs P30, ***$p = 0.0186$**; vs P30 2x, ***$p = 0.0187$**							
% CB-IR RCs No TdT No EGFP	#1	34.7	22.5	26.2	23.1	36.5	42.6	20.4
	#2	33.5	28.7	28.1	25.1	31.2	25.5	29.2
	#3	41.5	40.0	23.8	12.4	23.5	41.2	35.0
	**Average ± S.D.**	**36.6 ±4.3**	**30.4 ±8.9**	**26.1 ±2.1**	**20.2 ±6.9**	**30.4 ±6.5**	**36.4 ±9.5**	**28.2 ±7.4**
	One-way ANOVA: $F(6,14) = 2.071$ $p = 0.1228$							

<u>Supplemental Table 12</u> (refer to graphs in Fig 5C and S6)

Non-CB-IR V1 cells targeted by *Calb1/En1* intersection after TMP injections in *Calb1* dgCre/+ :: *En1*-Flpo/+ :: R26 FLTG animals

(data are average numbers of cells per RCA, 8-22 RCAs analyzed per animal; all animals were analyzed at P60 unless otherwise specified)

	TMP injection → analysis / Animal #	P60	P15 → P21	P15 2x	P21	P21 2x	P30	P30 2x
RCA	#1	0.0	1.8	0.1	0.7	0.7	0.7	1.2
	#2	0.0	0.6	0.1	0.1	1.0	0.1	0.3
	#3	0.4	0.2	0.8	0.2	0.0	0.1	0.1
	**Average ± S.D.**	**0.1 ±0.2**	**0.8 ±0.8**	**0.3 ±0.4**	**0.3 ±0.3**	**0.6 ±0.5**	**0.3 ±0.3**	**0.5 ±0.6**
	One-way ANOVA: F (6,14) = 0.6690 p = 0.6764							
Dorsal to RCA	#1	0.0	5.8	2.5	0.2	0.8	2.3	7.4
	#2	0.1	3.0	1.1	0.2	0.4	0.2	0.8
	#3	2.4	1.8	6.3	3.7	0.3	0.1	0.1
	**Average ± S.D.**	**0.8 ±1.4**	**3.5 ±2**	**3.3 ±2.7**	**1.4 ±2**	**0.5 ±0.3**	**0.9 ±1.3**	**2.8 ±4**
	One-way ANOVA: F (6,14) = 0.9727 p = 0.4784							

Supplemental Table 13

Genotyping probes and PCR primers used in this study

Name	Transnetyx probes	PCR primers
En1-Cre	En1-2 WT, CRE	N/A (was not genotyped by PCR)
Mafb-GFP	Mafb-3 WT, eGFP	5'-AGCAGCCGATTGTCTGTTGTGCCCAGTCAT-3' (KI, antisense); 5'-CCATCCAGTACAGGTCCTCG-3' (shared); 5'-TGAGCATGGGGCAAGAGCTG-3' (WT, sense)
Calb1-dgCre	Calb1-3 WT, CRE	JAX#023531 protocol (primers 19657, 19658, 19659)
Pvalb-Flpo	Pvalb-1 WT, Flpo	JAX#022730 protocol (primers 16211, 17564, 17566)
En1-Flpo	En1-2 WT, Flpo	5'-GAGAGCGAGATTTGCTCCAC-3' (KI, forward); 5'-GTTCACGATGTCGAAGCTCA03 (KI, reverse)
Ai9 R26 *lsl-tdT*	ROSA WT, tdRFP	JAX#007909 protocol (primers oIMR9020, oIMR9021, oIMR9103, oIMR9105)
R26 *RCE:dual*	ROSA WT, eGFP	N/A (was not genotyped by PCR)
R26-FLTG	ROSA WT, tdRFP	N/A (was not genotyped by PCR)
R26 *RCE-fsf-GFP*	N/A	5'-CCCAAAGTCGCTCTGAGTTGTTATC-3' (shared) 5'-GAAGGAGCGGGAGAAATGGATATG-3' (WT) 5'-CCAGGCGGGCCATTTACCGTAAG-3' (KI)

Supplemental Table 14

Primary antibodies used in the study

Antigen	Host	Immunogen	Source	Dilution	Specificity
Calbindin	Rabbit	recombinant rat calbindin D-28k	Swant CB-38a	1:500-1:1000	KO-verified[1,2] and by manufacturer
Parvalbumin	Chicken	Bacterial GST fusion protein with full length parvalbumin	Covance (AP37-47); gift by George Mentis	1:10,000	Validated against PV-Cre reporter expression[3]
Calretinin	Rabbit	Recombinant human calretinin containing a 6-his tag at the N-terminal	Swant 7699/4	1:1000	KO-verified by manufacturer; validated against antiserum and protein[4]
dsRed	Rabbit	Recombinant ZsYellow Living Colors Anti-RCFP Crossreacts with DsRed	Clontech 632496	1:1000	Verified in animals not expressing the fluorescent reporter
RFP	Mouse	Red Fluorescent Protein fusion protein derived from *Discosoma*	Rockland 200-301-379	1:1000	Verified in animals not expressing the fluorescent reporter
mCherry	Goat	Purified recombinant peptide produced in *E. coli*	MyBioSource.com MBS448050	1:250	Verified in animals not expressing the fluorescent reporter
GFP	Chicken	Recombinant GFP	Aves Labs GFP-1020	1:1000	Verified in animals not expressing the fluorescent reporter
GFP	Sheep	Green fluorescent protein from *Aequorea victoria*	Biogenesis 4745-1051	1:1000	Verified in animals not expressing the fluorescent reporter
GFP	Rabbit	Green fluorescent protein from *Aequorea victoria*	Invitrogen A-11122	1:2000	Verified in animals not expressing the fluorescent reporter
En1	Guinea Pig	Full length *Xenopus* Engrailed1 sequence fused to GST	Jessel Lab/HHMI CU	1:8000	KO-verified in tissue
NeuN	Mouse	purified cell nuclei from mouse brain	EMD Millipore MAB377	1:250-1:500	Recognizes the DNA-binding, neuron-specific protein fox3
VAChT	Guinea Pig	Recombinant protein corresponding to AA 475 to 530 from rat VAChT	Synaptic Systems 139 105	1:200	Same staining as a related antibody against same antigen that was KO-verified[5]
Gephyrin	Mouse	Native Protein corresponding to AA 1 to 768 from rat Gephyrin, clone 7a	Synaptic Systems 147 021	1:50	KO-verified; specific to the brain-specific 93 kDa splice variant phosphorylated at Ser-270[6]

1. Siembab, V. C., Gomez-Perez, L., Rotterman, T. M., Shneider, N. A. & Alvarez, F. J. Role of primary afferents in the developmental regulation of motor axon synapse numbers on Renshaw cells. *The Journal of comparative neurology* **524**, 1892-1919, doi:10.1002/cne.23946 (2016).
2. Airaksinen, M. S. *et al.* Ataxia and altered dendritic calcium signaling in mice carrying a targeted null mutation of the calbindin D28k gene. *Proc Natl Acad Sci U S A* **94**, 1488-1493, doi:10.1073/pnas.94.4.1488 (1997).
3. Fletcher, E. V. *et al.* Reduced sensory synaptic excitation impairs motor neuron function via Kv2.1 in spinal muscular atrophy. *Nat Neurosci* **20**, 905-916, doi:10.1038/nn.4561 (2017).
4. Gander, J. C. *et al.* The calcium-binding protein calretinin-22k, an alternative splicing product of the calretinin gene is expressed in several colon adeno carcinoma cell lines. *Cell Calcium* **20**, 63-72, doi:10.1016/s0143-4160(96)90051-2 (1996).
5. Kolisnyk, B. *et al.* Forebrain deletion of the vesicular acetylcholine transporter results in deficits in executive function, metabolic, and RNA splicing abnormalities in the prefrontal cortex. *J Neurosci* **33**, 14908-14920, doi:10.1523/jneurosci.1933-13.2013 (2013).
6. Feng, G. *et al.* Dual requirement for gephyrin in glycine receptor clustering and molybdoenzyme activity. *Science* **282**, 1321-1324, doi:10.1126/science.282.5392.1321 (1998).

Supplemental Table 15

Fluorochrome conjugated secondary antibodies used in this study.

Secondary antibodies	Source	Catalog Number
anti-chicken FITC IgY	Jackson Immunoresearch	703-095-155
anti-goat Cy3 IgG	Jackson Immunoresearch	705-165-147
anti-guinea pig Alexa Fluor® 647 IgG	Jackson Immunoresearch	706-605-148
anti-mouse Cy3 IgG	Jackson Immunoresearch	715-165-150
anti-mouse Cy5 IgG	Jackson Immunoresearch	715-175-150
anti-rabbit Alexa Fluor® 647 IgG	Jackson Immunoresearch	711-605-152
anti-chicken FITC IgY	Jackson Immunoresearch	703-095-155
anti-rabbit Alexa Fluor® 555 IgG	Jackson Immunoresearch	711-165-152
anti-rabbit Alexa Fluor® 647 IgG	Jackson Immunoresearch	711-605-152
anti-rabbit FITC IgG	Jackson Immunoresearch	711-095-152
anti-rabbit DyLight™ 405 IgG	Jackson Immunoresearch	711-475-152
anti-rabbit Alexa Fluor® 488 IgG	Jackson Immunoresearch	706-165-148
anti-guinea pig Cy3 IgG	Jackson Immunoresearch	711-545-152

Supplemental Table 16

Details of immunolabeling combinations used in figures and analyses

Figure	Animal	Primary antibodies	Secondary antibodies	Quantification
1B,C	*En1*-Cre/+ :: *Mafb*-GFP/+ :: Ai9 R26 *lsl-tdT/+*	P5: Calbindin (Rb), GFP (C)<br><br>P15: Calbindin (Rb), GFP (C)<br><br>6mo: Calbindin (Rb), GFP (C), RFP (M)	anti-chicken FITC IgY, anti-rabbit DyLight™ 405 IgG<br><br>anti-chicken FITC IgY, anti-rabbit Alexa Fluor® 647 IgG<br><br>anti-chicken FITC IgY, anti-rabbit Alexa Fluor® 647 IgG, anti-mouse Cy3 IgG	All *En1*-tdT cells were counted and marked for *Mafb*-GFP or CB-IR. In the RCA, any additional Mafb-GFP or CB-IR cells were counted.
2B (top), 2C	*Calb1*-dgCre/+ :: *Mafb*-GFP/+ :: Ai9 R26 *lsl-tdT/+*	Calbindin (Rb), GFP (C)	anti-chicken FITC IgY, anti-rabbit Alexa Fluor® 647 IgG	In the RCA: all cells expressing *Calb1*-tdT, *Mafb*-GFP, or CB-IR were counted.
2B (bottom), 2D,E	*Calb1*-dgCre/+ :: Ai9 R26 *lsl-tdT/+*	Calbindin (Rb)	anti-rabbit Alexa Fluor® 647 IgG *or* anti-rabbit FITC IgG	In the RCA: all cells expressing *Calb1*-tdT or CB-IR were counted.
2F	*Calb1*-dgCre/+ :: Ai9 R26 *lsl-tdT/+*	Calretinin (Rb)	anti-rabbit FITC IgG	In the RCA: all cells expressing *Calb1*-tdT were counted and marked for CR-IR.
2G	*Calb1*-dgCre/+ :: Ai9 R26 *lsl-tdT/+*	Parvalbumin (C), ± Calbindin (Rb)	anti-chicken FITC IgY, anti-rabbit Alexa Fluor® 647 IgG	In the RCA: all cells expressing *Calb1*-tdT were counted and marked for PV-IR. In animals co-immunostained for calbindin, all CB-IR cells in the RCA were counted and marked for PV-IR.
3B,C	*Calb1*-dgCre/+ :: *Pvalb*-Flpo/+ :: R26 *RCE:dual/+*	Calbindin (Rb), GFP (C)	anti-rabbit Alexa Fluor® 555 IgG, anti-chicken FITC IgY	All *Calb1*/*Pvalb*-EGFP cells were counted and marked for CB-IR. In the RCA, any additional CB-IR cells were counted.
4B	*En1*-Flpo :: R26 *RCE-fsf-GFP*	GFP (Rb), En1 (GP)	anti-rabbit Alexa Fluor® 488 IgG, anti-guinea pig Cy3 IgG	N/A
5B,C	*Calb1* dgCre/+ :: *En1*-Flpo/+ :: R26-FLTG	Calbindin (Rb), GFP (C), mCherry (Gt)	anti-chicken FITC IgY, anti-rabbit Alexa Fluor® 647 IgG, anti-goat Cy3 IgG	All *En1*/*Calb1*/-EGFP cells were counted and marked for CB-IR.<br><br>In the RCA, any additional CB-IR cells were counted.
5D-F	*En1*-Flpo/+ :: R26-FLTG	Calbindin (Rb), Gephyrin (M), mCherry (Gt)	anti-rabbit FITC IgG, anti-mouse Cy5 IgG, anti-goat Cy3 IgG	All ventral horn CB-IR with large cell clusters were counted and scored for tdT expression
5G,H	*En1*-Flpo/+ :: R26-FLTG	Calbindin (Rb), VAChT (GP), mCherry (Gt)	anti-rabbit FITC IgG, anti-guinea pig Cy5/647 IgG, anti-goat Cy3 IgG	All ventral horn CB-IR with high densities of VAChT synapse on their dendrites were counted and scored for tdT expression
6	Calb1 dgCre/+ :: En1-Flpo/+ :: R26-FLTG	GFP (C), NeuN (M), mCherry (Gt)	anti-chicken FITC IgY, anti-mouse Cy5 IgG, anti-goat Cy3 IgG	Brains; N/A
6	*Calb1*-dgCre/+ :: *Pvalb*-Flpo/+ :: R26 *RCE:dual/+*	GFP (C)	anti-chicken FITC IgY	Brains; N/A
7	*Calb1*-dgCre/+ :: *Pvalb*-Flpo/+; AAV9-DC-EYFP	Calbindin (Rb), GFP (C), NeuN (M)	anti-rabbit Alexa Fluor® 555 IgG, anti-chicken FITC IgY, anti-mouse Cy5 IgG	All EYFP+ cells were counted and marked for CB-IR. In the RCA, any additional CB-IR cells were counted.
S1A	Various	Calbindin (Rb)	anti-rabbit Alexa Fluor® 647 IgG *or* anti-rabbit FITC IgG	N/A
S1B	*Calb1*-dgCre/+ :: Ai9 R26 *lsl-tdT/+*	N/A	N/A	N/A
S5B	*Calb1*-dgCre/+ :: *Pvalb*-Flpo/+ :: R26 *lsl-tdT*/ *RCE:dual*	Calbindin (Rb), GFP (C)	anti-chicken FITC IgY, anti-rabbit Alexa Fluor® 647 IgG	All *Calb1*/*Pvalb*-EGFP cells were counted and marked for CB-IR and *Calb1*-tdT. In the RCA, any additional CB-IR or *Calb1*-tdT cells were counted.

Rb, Rabbit; C, Chicken; Gt, Goat; M, Mouse; GP, Guinea Pig
