# Supplementary material for: Genetic targeting of adult Renshaw cells using a Calbindin 1 destabilized Cre allele for intersection with Parvalbumin or Engrailed1
Source: Sci Rep. 2021 Oct 6;11:19861. doi: 10.1038/s41598-021-99333-6 (PMC8494874; doi:10.1038/s41598-021-99333-6)
Supplement: Supplementary file 2 — Supplementary Information 2. [file 41598_2021_99333_MOESM2_ESM.pdf]

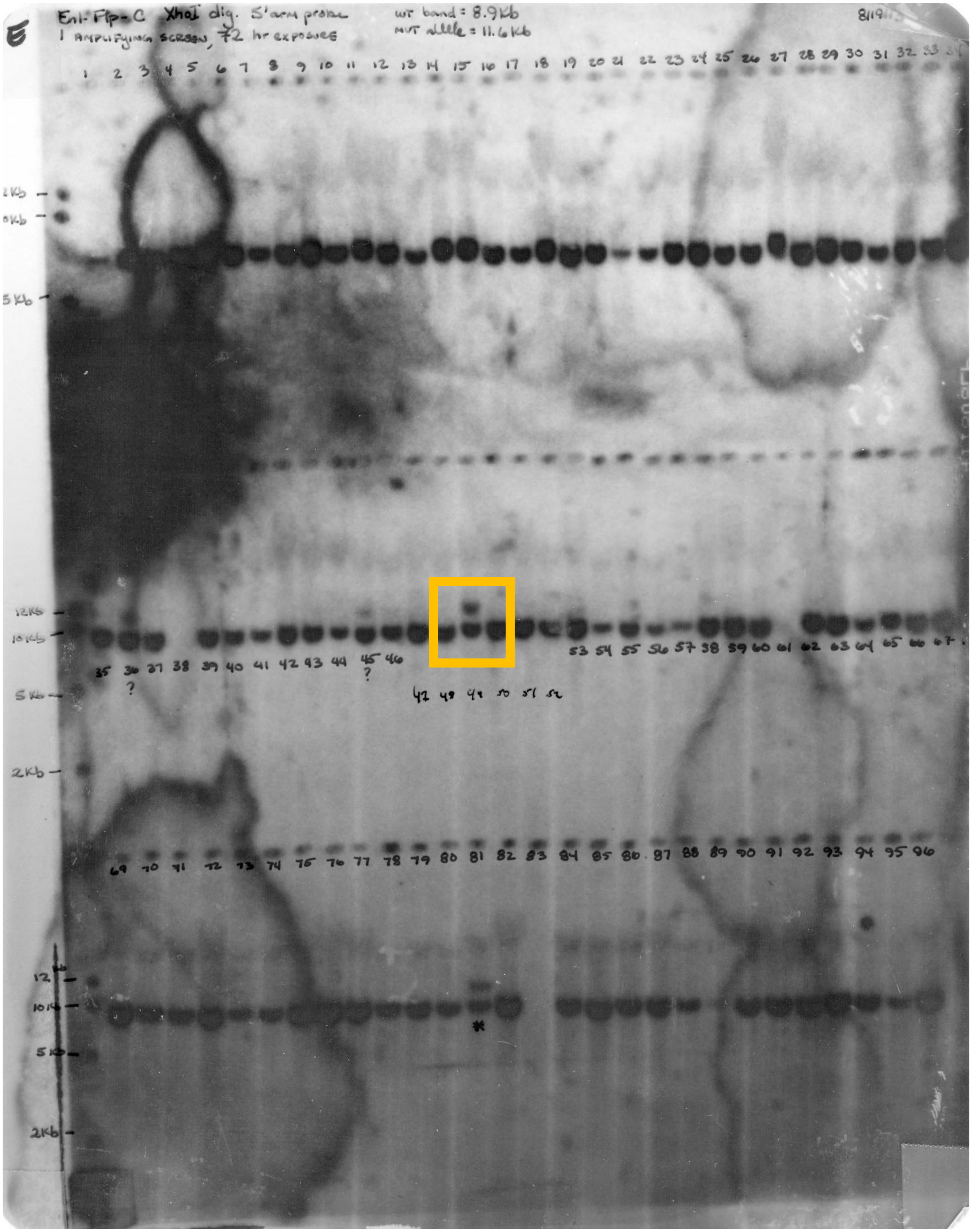

Original lab-annotated Southern blot gel of XhoI-digested genomic DNA from 96 ES cell clones. ES clones were screened with a 5' probe external to the targeting vector that identifies an 8.9 kb wild-type fragment and an 11.9 kb knock-in fragment in those lines containing the construct. Left lane show markers at 12, 10 and 5 Kb. Samples 49 and 81 show presence of the WT and knock-in fragment. The area inside the box was used in Figure 4A. Positive clones were used for injection in blastocysts.

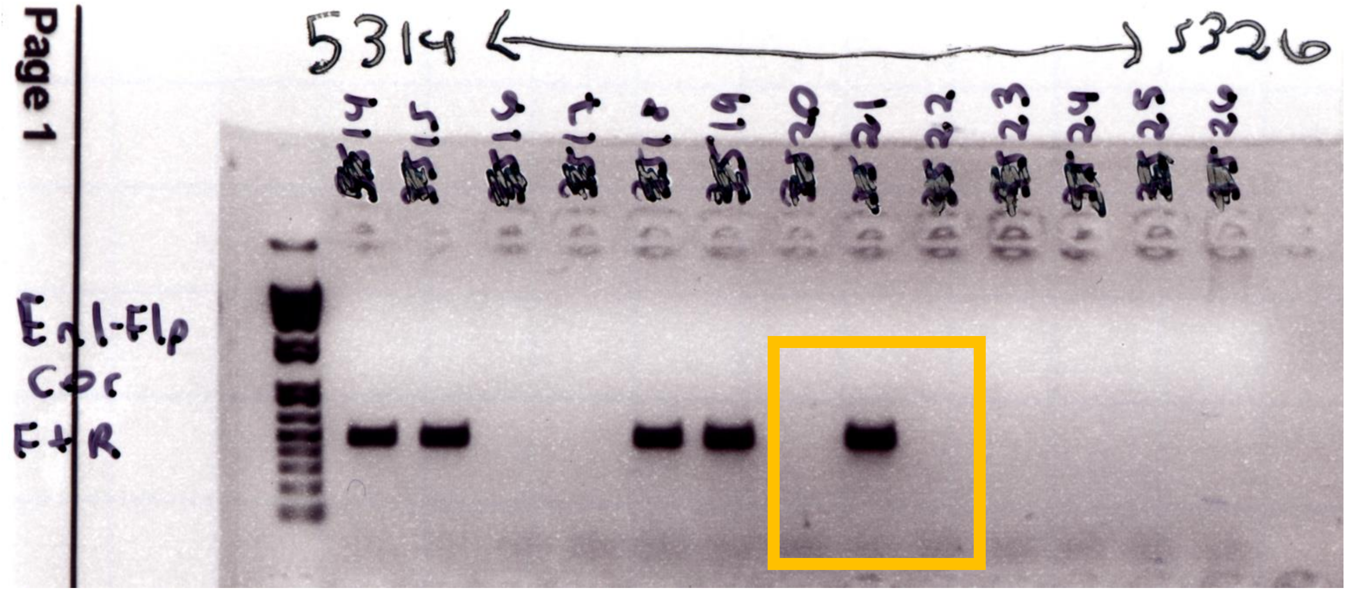

Original lab-annotated PCR gel using 5’-GAGAGCGAGATTTGCTCCAC-3’ (knock-in, forward) and 5’-GTTCACGATGTCGAAGCTCA-3 (knock-in, reverse) identifies a 484 bp band corresponding to the *En1::Flpo* allele. Each lane is a sample from a different animal. Animals 5314, 5315, 5318, 5319, and 5321 show the presence of the knock-in allele. The area inside the box was used in the illustration in Figure 4A. Animals carrying the knock-in *En1::Flpo* allele were used to start the colony.
